# Metabolic Saliency as KL-Divergence Estimator: Information-Geometric Attribution of Systemic Stress in JSE Equity Network

**DOI:** 10.3390/e28050559

**Published:** 2026-05-15

**Authors:** Ntebogang Dinah Moroke

**Affiliations:** Department of Statistics, Faculty of Economic and Management Sciences, Mafikeng Campus, North-West University, Private Bag X2046, Mmabatho 2735, South Africa; ntebo.moroke@nwu.ac.za

**Keywords:** metabolic saliency, Kullback–Leibler divergence, fisher information geometry, transfer entropy, systemic risk, interpretable deep learning, Johannesburg Stock Exchange, sustainable development goals, emerging markets

## Abstract

The attribution of systemic financial stress to specific market sectors requires metrics that are faithful to the model’s computations, statistically consistent, and connected to a physically meaningful measure of directed information flow. This paper addresses all three requirements through information geometry, contributing to SDGs 7, 8, 9, and 17 through an entropic causal chain linking energy infrastructure failure to financial market stress. We conjecture and empirically verify the **Entropy–Saliency Equivalence**: Metabolic Saliency is an asymptotically unbiased estimator of the local Kullback–Leibler divergence between stressed and resting sector return distributions, with bias decaying at a parametric rate under Gaussian regularity conditions. The finite-sample bias–variance decomposition of the Kraskov–Stögbauer–Grassberger transfer entropy estimator is derived, establishing a minimax-optimal convergence rate. A novel metric, the **Spatio-Temporal Information Flux** (STIF), quantifies directed inter-sector stress transmission in bits per trading day, providing a bootstrap-calibrated audit trail aligned with the South African Financial Sector Regulation Act and MiFID II. Empirical validation on the JSE canonical panel (87 securities, 2857 trading days, 2015–2026) with Eskom load-shedding stages as exogenous stress injectors confirms the equivalence ($R^{2}=0.810$, $\hat{\rho }=0.90$), with walk-forward $R^{2}=0.789$ and placebo $R^{2}=0.081$ ruling out estimation artefacts. The energy sector is identified as the primary stress transmitter during Stage 4+ Eskom events (STIF rising from 0.14 to 0.43 bits/day, directional asymmetry ratio 4.7). Robustness checks confirm stability across non-Gaussian securities and rolling transfer entropy windows.

## 1. Introduction

Financial markets are complex adaptive systems in which stress propagates through interconnected institutions via contagion channels [1], creating systemic risk that is difficult to attribute to specific sectors or shocks [2]. Identifying which sector originates a stress event, and quantifying how much information it transmits to downstream sectors, is both a practical requirement for risk officers and a legal requirement under the South African Financial Sector Regulation Act [3] and the European Union’s MiFID II directive [4], which mandate that models used in licensed financial services be explainable and auditable.

The most widely used attribution methods address this need only partially. SHAP values [5] and LIME [6] provide local linear approximations that are not guaranteed to be faithful to the model’s true computational graph and can be adversarially manipulated to produce misleading explanations [7,8]. Integrated Gradients [9] satisfies stronger axiomatic guarantees (Sensitivity and Implementation Invariance) but relies on a fixed zero-input baseline that conflates “zero input” with “market at rest”, a distinction that carries material consequences when the resting state of a market is characterised by a non-zero empirical return distribution. None of these methods provides a directional, unit-bearing measure of inter-sector stress transmission.

Metabolic Saliency $S_{\mathrm{ms}}\left(i,t\right)$, introduced in the companion paper of this series (Moroke [10], under review), is an architecturally intrinsic attribution metric derived from the Jacobian of the Power Mapping Network (PMNet) output with respect to the input voxel, which is weighted by pairwise transfer entropy [11] between JSE sectors. The metric is faithful by construction (the Jacobian is the exact derivative of the model’s forward pass) and empirically stable under retraining (cf. the companion paper; Moroke, under review). All definitions required for this paper are stated in full in Section 3 and Section 4; access to the companion paper is not required. An initial empirical correlation of $\hat{\rho }=0.73$ between $S_{\mathrm{ms}}$ and entropy production during Eskom stress events suggests the metric tracks market stress, but provides no convergence guarantee. This paper establishes that guarantee.

The central theoretical contribution is the **Entropy–Saliency Equivalence** (Conjecture 1): under regularity conditions on the PMNet output distribution, $S_{\mathrm{ms}}\left(i,t\right)$ is an asymptotically unbiased estimator of the local Kullback–Leibler (KL) divergence $\mathrm{KL}(q_{t}^{\left(i\right)}∥q_{0}^{\left(i\right)})$ between the stressed and resting sector return distributions. This result is established as an empirically verified conjecture using the information geometry of exponential families [12] and the Fisher score representation of the PMNet Jacobian [13]. The convergence rate is characterised via the Cramér–Rao lower bound [14], and the finite-sample bias of the KSG transfer entropy estimator [15] is analysed via a minimax rate theorem.

### 1.1. Positioning Within the Literature

Information-geometric approaches to neural network attribution have a growing literature. Kakade [13] showed that the natural gradient in function space is related to the Fisher information of the model’s output distribution, a result that underlies the connection between the PMNet Jacobian and the KL divergence established here. Martens [16] developed Kronecker-factored approximations to the Fisher information matrix for deep networks, demonstrating the practical value of Fisher-geometric methods in large-scale architectures. The present contribution makes this connection explicit and statistically rigorous for the GWS-STNet architecture, and extends it to financial stress attribution via the Schreiber–Barnett equivalence between transfer entropy and Granger causality [17].

In the financial econometrics literature, transfer entropy has been applied to measure information flow between financial markets by Kwon and Yang [18], Dimpfl and Peter [19], and Sandoval [20]. Financial contagion and systemic risk propagation have been studied through network-theoretic lenses by Allen and Gale [1], who showed that liquidity shocks propagate through balance-sheet linkages to create systemic fragility, and through empirical stylised facts by Cont [2]. At the systemic risk measurement level, the Diebold–Yilmaz spillover index [21] decomposes forecast error variance to measure directional spillovers across institutions, and Adrian and Brunnermeier’s CoVaR [22] measures tail-risk contributions of individual institutions to system-wide stress. These measures are informative but share a common limitation: they do not connect to the internal computations of a predictive model and cannot provide a model-faithful attribution of stress to its causal antecedents. The present contribution addresses this gap by embedding directional stress transmission within a convergent neural attribution framework and establishing its convergence properties. These papers treat transfer entropy or contagion as a descriptive tool; the present contribution embeds transfer entropy within a neural network attribution framework and establishes its convergence properties in this setting. The nearest related work is that of Bianchi et al. [23], who apply Fisher information to bond risk premia, and Gu et al. [24], who apply information-theoretic methods to equity factor selection. It neither establishes a convergence rate for a Jacobian-based attribution metric nor validates the framework in an infrastructure-constrained emerging market panel.

### 1.2. Contributions

This paper makes four contributions:

(i) **Entropy–Saliency Equivalence.** We conjecture and empirically verify that $S_{\mathrm{ms}}\left(i,t\right)$ is an asymptotically unbiased estimator of $\mathrm{KL}(q_{t}^{\left(i\right)}∥q_{0}^{\left(i\right)})$, with bias decaying at rate $O(T_{0}^{-1})$ under Gaussian regularity conditions on the PMNet output distribution. The connection between a Jacobian-based attribution metric and a KL divergence with an explicit convergence rate has not previously been established in a financial panel setting.
(ii) **KSG bias–variance decomposition.** We characterise the finite-sample properties of the KSG estimator of transfer entropy as used in $S_{\mathrm{ms}}$, establishing a minimax-optimal convergence rate and providing explicit block bootstrap confidence intervals for the saliency weights.
(iii) **Spatio-Temporal Information Flux (STIF).** We propose STIF as an evaluation metric quantifying sector-level directed information flow in bits per trading day and establish its consistency under the same regularity conditions as the equivalence result.
(iv) **Regulatory audit protocol.** We demonstrate a three-step auditing procedure (Jacobian check, transfer entropy check, Eskom record verification) that provides a quantitative framework aligned with the interpretability requirements of the FSRA [3] and MiFID II [4].

### 1.3. Paper Organisation

Section 3 reviews the information-geometric framework and states all definitions required for the main results. Section 4 states and empirically verifies the Entropy–Saliency Equivalence. Section 5 analyses the KSG estimator and derives the bias–variance decomposition. Section 6 defines the STIF metric and establishes its consistency. Section 7 describes the empirical design. Section 8 presents the results. Section 9 discusses implications and limitations. Section 10 concludes.

## 2. Related Work and Literature Positioning

This paper draws on three research streams: (i) information geometry applied to neural networks; (ii) transfer entropy estimation for financial networks; (iii) statistical attribution methods for regulatory compliance. Table 1 maps the most closely related papers against the seven defining features of this paper.

### 2.1. Stream 1: Information Geometry and Neural Networks

The connection between neural network training and the geometry of the statistical manifold was established by Amari [12] through the natural gradient framework and later extended by Martens [16] to practical deep learning via Kronecker-factored approximations to the Fisher information matrix. Kakade [13] demonstrated that the natural gradient in function space coincides with the Fisher score, a result that underlies the Entropy–Saliency Equivalence of Section 4. The Cramér–Rao lower bound [14] provides the theoretical benchmark against which the asymptotic efficiency of Metabolic Saliency is measured (Theorem 1), and the exponential family regularity conditions that underpin its derivation are formalised by Brown [25].

The existing literature has not applied information geometry to neural network *attribution* in financial systems. Three specific gaps motivate this paper: (i) no existing paper establishes that a Jacobian-based attribution metric converges to a KL divergence with an explicit rate; (ii) no existing paper provides a minimax-optimal convergence rate for transfer entropy weights used in a neural attribution formula; (iii) no existing paper derives a formal audit protocol from first principles rather than a post hoc approximation for an emerging market context.

### 2.2. Stream 2: Transfer Entropy in Financial Networks

Transfer entropy was introduced to financial networks by Schreiber [11], and its equivalence with Granger causality for Gaussian variables was established by Barnett et al. [17]. Kwon and Yang [18] applied TE to measure directional information flow between stock market indices; Dimpfl and Peter [19] extended this to volatility spillovers across asset classes; Sandoval [20] constructed TE-based directed networks for global equity markets. Financial contagion and stress propagation in interconnected systems have been studied by Allen and Gale [1], providing the economic motivation for sector-level attribution in systemic risk contexts. The empirical systemic risk literature has developed several measures for quantifying stress transmission. The Diebold–Yilmaz spillover index [21] decomposes forecast error variance to measure directional spillovers across financial institutions at daily frequency. Adrian and Brunnermeier’s CoVaR (2016) measures the contribution of individual institutions to system-wide tail risk. These measures are informative but share a common limitation: they do not connect to the internal computations of a predictive model and cannot provide a model-faithful attribution of stress to its causal antecedents. The STIF metric addresses this gap by embedding directional stress transmission within a convergent neural attribution framework. The KSG estimator [15] is the standard non-parametric tool for TE estimation in continuous state spaces, and its consistency was established by Kozachenko and Leonenko [26]. The minimax-optimal convergence rate for *k*-NN density estimators in *d*-dimensional space, established by Devroye and Wagner [27] and synthesised by Biau and Devroye [28], provides the theoretical foundation for Theorem 2.

Deep neural models applied to JSE equity forecasting, including GPT-SNN-PPO and LSTM architectures [29], confirm that neural approaches outperform classical time-series methods on the same JSE panel, providing further motivation for model-faithful attribution of the neural predictions generated in this market. What this stream lacks, however, is a connection between TE estimation and neural network attribution: TE is used as a standalone descriptive tool rather than as a weighting scheme within a deep learning model whose convergence properties it affects. The present paper makes this connection explicit and characterises the implied finite-sample uncertainty in the attribution weights.

### 2.3. Stream 3: Attribution Methods for Regulatory Compliance

The regulatory demand for explainable AI in finance has generated substantial applied literature. SHAP [5] and LIME [6] are the dominant post hoc methods; their adversarial vulnerabilities have been documented by Adebayo et al. [8] and Slack et al. [7]. Integrated Gradients [9] satisfies the Sensitivity and Implementation Invariance axioms and provides a stronger gradient-based comparator, though it relies on a fixed zero-input baseline. Bianchi et al. [23] applied Fisher information to construct interpretable bond risk premia, the closest existing work to the approach taken here. Gu et al. [24] used information-theoretic factor selection for equity return prediction. In the cryptocurrency domain, Moroke [30] embedded free-energy efficiency bounds within a deep reinforcement learning portfolio management framework, demonstrating that information-geometric attribution extends beyond equity markets to asset classes with different microstructure properties. At the regulatory level, both MiFID II [4] and the South African FSRA [3] mandate model auditability without specifying a quantitative statistical standard for what “auditable” means.

The STIF metric and the three-step audit protocol of Section 6 address this gap by providing a unit-bearing, statistically consistent, directional measure of inter-sector stress attribution that is directly interpretable by non-technical stakeholders.

### 2.4. Literature Gap Map

**Synthesis.** The three streams converge on a common gap: Stream 1 provides the geometric machinery, but has not applied it to attribution in financial systems; Stream 2 uses transfer entropy as a descriptive tool, but has not embedded it within a convergent attribution framework; Stream 3 acknowledges regulatory requirements, but has not derived a quantitative standard from first principles. This paper addresses all three gaps simultaneously through the Entropy–Saliency Equivalence and the STIF metric.

**Table 1:** Literature gap map. Features: (F1) Convergence proof for attribution metric; (F2) KL-divergence connection to Jacobian; (F3) KSG bias–variance decomposition; (F4) Directed TE weighting in neural attribution; (F5) STIF metric in bits/day; (F6) Regulatory audit protocol from first principles; (F7) Emerging market (JSE-Eskom) validation. •: full contribution; ∘: partial; −: not applicable.

Paper	Journal	Stream	F1	F2	F3	F4	F5	F6	F7
Amari [12]	*Springer *	1	•	•	−	−	−	−	−
Martens [16]	*JMLR*	1	∘	∘	−	−	−	−	−
Kakade [13]	*NeurIPS*	1	∘	•	−	−	−	−	−
Cramér [14]	*Princeton UP*	1	•	−	−	−	−	−	−
Rao [31]	*Bull. Cal. Math*	1	•	−	−	−	−	−	−
Brown [25]	*IMS*	1	∘	∘	−	−	−	−	−
Schreiber [11]	*PRL*	2	−	−	−	•	−	−	−
Barnett et al. [17]	*PRL*	2	∘	−	−	•	−	−	−
Kraskov et al. [15]	*Phys. Rev. E*	2	−	−	∘	−	−	−	−
Biau & Devroye [28]	*Springer*	2	∘	−	•	−	−	−	−
Kwon & Yang [18]	*EPL*	2	−	−	−	∘	−	−	−
Dimpfl & Peter [19]	*SNDE*	2	−	−	−	∘	−	−	−
Sandoval [20]	*Entropy*	2	−	−	−	∘	−	−	−
Allen & Gale [1]	*JPE*	2	−	−	−	−	−	−	∘
Lundberg & Lee [5]	*NeurIPS*	3	−	−	−	−	−	∘	−
Ribeiro et al. [6]	*KDD*	3	−	−	−	−	−	∘	−
Sundararajan et al. [9]	*ICML*	3	−	−	−	−	−	∘	−
Adebayo et al. [8]	*NeurIPS*	3	−	−	−	−	−	−	−
Slack et al. [7]	*AIES*	3	−	−	−	−	−	−	−
Bianchi et al. [23]	*RFS*	3	∘	∘	−	−	−	∘	−
Gu et al. [24]	*RFS*	3	−	−	−	−	−	∘	−
MiFID II & FSRA [3,4]	Legislation	3	−	−	−	−	−	∘	−
**Shoko et al. [29]**	*Rom. J. Econ.*	2	−	−	−	∘	−	−	•
Adrian & Brunnermeier [22]	*AER*	2	−	−	−	−	−	−	∘
Moroke [30]	*Risks*	3	−	∘	−	−	−	∘	∘
**This paper**	*Entropy*	1 + 2 + 3	•	•	•	•	•	•	•

## 3. Information-Geometric Preliminaries

Information geometry provides the natural mathematical setting for this paper. The central idea, developed by Amari [12], is that a parametric family of probability distributions forms a Riemannian manifold equipped with the Fisher information metric, and that operations on distributions such as KL divergence and score functions have natural geometric interpretations on this manifold. This section establishes the three objects required for the main result in Section 4: the statistical manifold on which the PMNet output distribution lives, the Fisher metric that governs its geometry, and the resting and stressed sector-level distributions whose KL divergence Metabolic Saliency estimates.

### 3.1. Statistical Manifold and Fisher Metric

**Definition** **1** (Statistical manifold)**.** *Let $\Theta \subseteq R^{p}$ be a parameter space. A* statistical manifold *is a family of probability density functions S={p(·;θ):θ∈Θ} on R satisfying the following regularity conditions [12]: (i) p(·;θ) is smooth in θ; (ii) the support does not depend on θ; (iii) the score function $l_{\theta }\left(x\right)=\nabla_{\theta }\log p\left(x;\theta \right)$ has zero mean and finite second moment for all θ∈Θ*.

**Definition** **2** (Fisher information matrix)**.** *The* Fisher information matrix *at θ is*

(1) $$F\left(\theta \right)\;=\;E_{\theta }\;\left[l_{\theta }\left(X\right)l_{\theta }{\left(X\right)}^{⊤}\right]\;=\;-E_{\theta }\;\left[\nabla_{\theta }^{2}\log p\left(X;\theta \right)\right]\;\in \;R^{p\times p}.$$

*The Fisher matrix defines a Riemannian metric on S [31]: for a smooth curve θ(t) on S, the length element is $ds^{2}=\dot{\theta }{\left(t\right)}^{⊤}F\left(\theta \left(t\right)\right)\dot{\theta }\left(t\right)\;dt^{2}$. It also defines the natural gradient, which measures the steepest descent in the space of distributions rather than in the Euclidean parameter space [12,13]*.

**Lemma** **1** (KL divergence and Fisher metric)**.** *For distributions p(·;θ) and $p(\cdot ;\theta_{0})$ in a regular statistical manifold, the KL divergence admits the second-order approximation [12]:*

(2) $$\mathrm{KL}\;\left(p\left(\cdot ;\theta \right)\;∥\;p\left(\cdot ;\theta_{0}\right)\right)\;=\;\frac{1}{2}{\left(\theta -\theta_{0}\right)}^{⊤}F\left(\theta_{0}\right)\;\left(\theta -\theta_{0}\right)+O\;\left({∥\theta -\theta_{0}∥}^{3}\right).$$

**Proof.**  Taylor-expand $\mathrm{KL}(p\left(\cdot ;\theta \right)∥p\left(\cdot ;\theta_{0}\right))$ around $\theta =\theta_{0}$. The zero-order term vanishes since KL(p∥p)=0. The first-order term vanishes because the gradient of KL at $\theta =\theta_{0}$ equals the expectation of the score function under $p(\cdot ;\theta_{0})$, which is zero by the regularity conditions of Definition 1. The second-order coefficient is $\frac{1}{2}\nabla_{\theta }^{2}\mathrm{KL}{|}_{\theta =\theta_{0}}=\frac{1}{2}F\left(\theta_{0}\right)$ by the standard identity relating the KL Hessian to the Fisher matrix [12]. □

**Definition** **3** (Resting and stressed market distributions)**.** *For sector i∈N and trading day t∈T, define:*

- *The* resting distribution *$q_{0}^{\left(i\right)}$: the empirical return distribution of sector i over the training baseline (January 2015 to December 2018, $T_{0}=992$ days), estimated by kernel density estimation [32] with Gaussian kernel and bandwidth $h_{0}=1.06{\hat{\sigma }}_{i}T_{0}^{-1/5}$ (Silverman’s rule of thumb)*.
- *The* stressed distribution *$q_{t}^{\left(i\right)}$: the empirical return distribution of sector i over a rolling window of τ=22 trading days ending at day t, estimated by the same kernel density estimator with bandwidth $h_{t}=1.06{\hat{\sigma }}_{i,t}\tau^{-1/5}$. The window τ=22 corresponds to one calendar month of trading days, balancing responsiveness to stress events against estimation stability*.

*The* local KL divergence *at time t for sector i is*

(3) $$\Delta_{t}^{\left(i\right)}\;:=\;\mathrm{KL}\;\left(q_{t}^{\left(i\right)}\;∥\;q_{0}^{\left(i\right)}\right)\;=\;\int p\left(x;\theta_{t}^{\left(i\right)}\right)\log\frac{p(x;\theta_{t}^{\left(i\right)})}{p(x;\theta_{0}^{\left(i\right)})}\;dx.$$

*High values of $\Delta_{t}^{\left(i\right)}$ indicate that sector i’s return distribution has deviated substantially from its resting state, providing the information-geometric signature of metabolic stress. Low values indicate that the sector is operating within its historical range*.

**Remark** **1.** *Under the Gaussian approximation $q_{t}^{\left(i\right)}=N\left(\mu_{t}^{\left(i\right)},\sigma_{t}^{2(i)}\right)$ and $q_{0}^{\left(i\right)}=N\left(\mu_{0}^{\left(i\right)},\sigma_{0}^{2(i)}\right)$, the KL divergence reduces to the closed form [33]:*

(4) $$\Delta_{t}^{\left(i\right)}\;=\;\frac{{\left(\mu_{t}^{\left(i\right)}-\mu_{0}^{\left(i\right)}\right)}^{2}}{2\sigma_{0}^{2(i)}}+\frac{\sigma_{t}^{2(i)}}{2\sigma_{0}^{2(i)}}-\frac{1}{2}-\frac{1}{2}\log\frac{\sigma_{t}^{2(i)}}{\sigma_{0}^{2(i)}}.$$

*This closed form requires only the rolling mean and variance of sector i’s returns and is used in the empirical computations of Section 7. The equivalence result of Conjecture 1 holds for the general non-Gaussian case; the Gaussian form is used as the empirical implementation for computational tractability, with robustness to non-Gaussian securities verified in Section 8.9*.

### 3.2. Power Mapping Network and Metabolic Saliency

The PMNet $P:R^{d_{4}}\to R^{N}$ is a three-layer spectrally normalised multilayer perceptron with GELU activations (full architectural details in the companion paper; Moroke, under review):

(5) $$P\left(z\right)=W_{3}\;\phi \;\left(W_{2}\;\phi \;\left(W_{1}z+b_{1}\right)+b_{2}\right)+b_{3},$$

where ϕ is the GELU activation, $W_{k}$ are weight matrices satisfying $∥W_{k}{∥}_{2}\;\leq 1$ (spectral norm constraint), and the output dimension N=87 is the number of JSE sectors. The spectral normalisation ensures the composite map is non-expansive, which is the condition required for the topological stability results of the companion paper (Moroke, under review). The PMNet is trained to minimise the entropic loss

(6) $$L=\frac{1}{N}\sum_{i=1}^{N}\mathrm{KL}\;\left(p_{i}\;∥\;{\hat{p}}_{i}\right)+\lambda \sum_{k=1}^{3}{∥W_{k}∥}_{F}^{2},$$

where $p_{i}$ is the true return distribution and ${\hat{p}}_{i}$ is the PMNet-predicted distribution.

**Definition** **4** (Metabolic Saliency)**.** *The* Metabolic Saliency *of sector i at time t is*

(7) $$S_{\mathrm{ms}}\left(i,t\right)=\frac{\partial {\hat{m}}_{i}}{\partial x_{i,t}}\cdot \sum_{j\neq i}{\mathrm{TE}}_{j\to i}\left(l^{*}\right)\;e^{\alpha \;{\mathrm{TE}}_{j\to i}\left(l^{*}\right)},$$

*where $\partial {\hat{m}}_{i}/\partial x_{i,t}$ is the exact Jacobian of the PMNet output computed via backpropagation (no post hoc surrogate), ${\mathrm{TE}}_{j\to i}\left(l^{*}\right)$ is the KSG transfer entropy from sector j to sector i at optimal lag $l^{*}=3$, and α=0.1 is a temperature parameter. The transfer entropy weighting amplifies contributions from sectors that historically transmit the most directed information into sector i*.

### 3.3. Fisher Score Representation of the PMNet Jacobian

The key bridge between the PMNet Jacobian and information geometry is Lemma 2, which shows that the Jacobian approximates the Fisher score of the output distribution. This bridge is used as a heuristic in the derivation of Conjecture 1; the empirical validation of Section 8 constitutes the primary evidence for the result.

**Lemma** **2** (Fisher score representation of the Jacobian)**.** *Let $P:R^{d_{4}}\to R^{N}$ be the PMNet defined in Equation (5), and suppose the residuals ${\hat{m}}_{i}-m_{i}$ follow a distribution in a regular exponential family [25] with natural parameter $\theta_{t}^{\left(i\right)}$. Then the Jacobian of the PMNet output satisfies*

(8) $$\frac{\partial {\hat{m}}_{i}}{\partial x_{i,t}}\;=\;F{\left(\theta_{t}^{\left(i\right)}\right)}^{-1}\;l_{\theta_{t}^{\left(i\right)}}\left(x_{i,t}\right)\;+\;O\;\left({∥x_{i,t}-\mu_{i,t}∥}^{2}\right),$$

*where $l_{\theta_{t}^{\left(i\right)}}\left(x\right)=\nabla_{\theta }\log p\left(x;\theta_{t}^{\left(i\right)}\right)$ is the score function and $\mu_{i,t}=E_{q_{t}^{\left(i\right)}}\left[X\right]$*.

**Proof.**  For a PMNet trained to minimise the entropic loss (6), the first-order optimality condition at the minimum requires $\partial L/\partial {\hat{m}}_{i}=0$, which by the implicit function theorem and the chain rule gives $\partial {\hat{m}}_{i}/\partial x_{i,t}=-H_{x}^{-1}H_{\hat{m}}$, where $H_{x}$ and $H_{\hat{m}}$ are mixed second derivatives of the loss with respect to input and output, respectively. For a loss in the exponential family [25], $-H_{\hat{m}}=F\left(\theta_{t}^{\left(i\right)}\right)$ and $H_{x}=F\left(\theta_{t}^{\left(i\right)}\right)l_{\theta_{t}^{\left(i\right)}}{\left(x_{i,t}\right)}^{-1}$ to first order in $x_{i,t}-\mu_{i,t}$. The $O({∥x_{i,t}-\mu_{i,t}∥}^{2})$ remainder is the second-order Taylor residual. □

## 4. The Entropy–Saliency Equivalence

This section contains the two main theoretical results of the paper. Section 4.1 states the Entropy–Saliency Equivalence (Conjecture 1), which establishes asymptotic unbiasedness of normalised Metabolic Saliency as an estimator of the local KL divergence $\Delta_{t}^{\left(i\right)}$, and derives the heuristic five-step derivation that motivates it. Section 4.2 establishes the asymptotic distribution and the Cramér–Rao lower bound on the variance of any unbiased estimator of $\Delta_{t}^{\left(i\right)}$.

### 4.1. Main Result

**Assumption** **1** (Regularity conditions)**.** *The following conditions hold throughout the paper:*

*(i)*  *The PMNet residual distribution $p(\cdot ;\theta_{t}^{\left(i\right)})$ belongs to a regular exponential family with sufficient statistic T(x) and natural parameter $\theta_{t}^{\left(i\right)}\in \Theta \subseteq R^{p}$ [25]. This is verified empirically in Section 7.2: the Gaussian AIC weight exceeds 0.5 for 71/87 JSE securities; the 16 exceptions are identified and shown not to affect the main result in Section 8.9*.
*(ii)*  *The Fisher information matrix $F(\theta_{t}^{\left(i\right)})$ is positive definite for all i∈N, t∈T, with $\lambda_{\min}\left(F\right)>0$ uniformly bounded away from zero. Empirically verified on the JSE training baseline: ${\hat{\lambda }}_{\min}=0.14$, range [0.07,0.31] across all 87 sectors (Table 2)*.
*(iii)*  *The transfer entropy ${\mathrm{TE}}_{j\to i}\left(l^{*}\right)$ is estimated on the training set only (January 2015 to December 2018) using the KSG estimator [15] with k=5 nearest neighbours, giving a consistent estimate [26]. The look-ahead-free constraint ensures no information from the evaluation period contaminates the saliency weights*.
*(iv)*  *The temperature parameter α>0 satisfies $\alpha <\alpha^{*}=\log\left(N\right)/\max_{j\neq i}{\mathrm{TE}}_{j\to i}\left(l^{*}\right)$, ensuring the exponential weights $e^{\alpha \;{\mathrm{TE}}_{j\to i}}$ are summable and dominated by the maximum TE. For the JSE panel, α=0.1 and log(87)/maxTE≈3.8, so the constraint is satisfied by a wide margin*.

**Comparison with competing methods.** Assumption 1(i) requires exponential family residuals. For reference, SHAP requires feature independence [34], which is violated in the JSE panel (sectors are strongly correlated); Integrated Gradients [9] requires path-connectedness of the input domain, which holds trivially. The exponential-family condition is therefore neither stronger nor weaker than the assumptions of the dominant competing methods; it is simply different in kind.

**Conjecture** **1** (Entropy–Saliency Equivalence)**.** *Under Assumption 1, the Metabolic Saliency $S_{\mathrm{ms}}\left(i,t\right)$ is an asymptotically unbiased estimator of the local KL divergence $\Delta_{t}^{\left(i\right)}$ scaled by the transfer-entropy normalisation constant $C_{\mathrm{TE}}^{\left(i\right)}=\sum_{j\neq i}{\mathrm{TE}}_{j\to i}\left(l^{*}\right)e^{\alpha \;{\mathrm{TE}}_{j\to i}\left(l^{*}\right)}$:*

(9) $$E\;\left[S_{\mathrm{ms}}\left(i,t\right)\right]\;=\;C_{\mathrm{TE}}^{\left(i\right)}\cdot \Delta_{t}^{\left(i\right)}\;+\;B_{i,t},$$

*where the bias term satisfies*

(10) $$|B_{i,t}|\;\leq \;\frac{C_{\mathrm{TE}}^{\left(i\right)}}{\lambda_{\min}\left(F\right)}\cdot \frac{K_{3}\left(\theta_{t}^{\left(i\right)}\right)}{T_{0}^{1/2}},$$

*with $K_{3}\left(\theta \right)=E_{\theta }\;{\left[{∥l_{\theta }\left(X\right)∥}^{3}\right]}^{1/3}$ the third Fisher moment. Under Gaussian regularity conditions, the bias tightens to $|B_{i,t}|=O\left(T_{0}^{-1}\right)$ (see Step 3a of the derivation below), and $S_{\mathrm{ms}}\left(i,t\right)/C_{\mathrm{TE}}^{\left(i\right)}$ is a consistent estimator of $\Delta_{t}^{\left(i\right)}$ as $T_{0}\to \infty$*.

Heuristic derivation.

The following five-step derivation uses the Fisher score approximation of Lemma 2 as a heuristic bridge between the Jacobian and the KL divergence. The empirical validation of Section 8 constitutes the primary evidence for the result.

**Step 1: Score decomposition.** By Definition 4,

$$S_{\mathrm{ms}}\left(i,t\right)\;=\;\frac{\partial {\hat{m}}_{i}}{\partial x_{i,t}}\cdot C_{\mathrm{TE}}^{\left(i\right)}.$$

Applying Lemma 2:

(11) $$S_{\mathrm{ms}}\left(i,t\right)\;=\;C_{\mathrm{TE}}^{\left(i\right)}\;F{\left(\theta_{t}^{\left(i\right)}\right)}^{-1}l_{\theta_{t}^{\left(i\right)}}\left(x_{i,t}\right)\;+\;R_{i,t},$$

where the remainder $R_{i,t}=O\left({∥x_{i,t}-\mu_{i,t}∥}^{2}\right)$.

**Step 2: Expectation of the score term.** Taking expectations under $q_{t}^{\left(i\right)}$ and using the mean–parameter correspondence for exponential families [25], $E_{q_{t}^{\left(i\right)}}\left[l_{\theta_{t}^{\left(i\right)}}\left(X\right)\right]=\theta_{t}^{\left(i\right)}-\theta_{0}^{\left(i\right)}$:

(12) $$E_{q_{t}^{\left(i\right)}}\;\left[F{\left(\theta_{t}^{\left(i\right)}\right)}^{-1}l_{\theta_{t}^{\left(i\right)}}\left(x_{i,t}\right)\right]\;=\;F{\left(\theta_{t}^{\left(i\right)}\right)}^{-1}\left(\theta_{t}^{\left(i\right)}-\theta_{0}^{\left(i\right)}\right).$$

**Step 3: Connection to the KL divergence.** By Lemma 1, $\nabla_{\theta_{t}}\Delta_{t}^{\left(i\right)}=F\left(\theta_{0}\right)\left(\theta_{t}-\theta_{0}\right)+O\left({∥\Delta \theta ∥}^{2}\right)$. Since $F\left(\theta_{t}\right)=F\left(\theta_{0}\right)+O\left(∥\Delta \theta ∥\right)$ by continuity of the Fisher matrix in a regular exponential family [25]:

(13) $$F{\left(\theta_{t}^{\left(i\right)}\right)}^{-1}\left(\theta_{t}^{\left(i\right)}-\theta_{0}^{\left(i\right)}\right)\;=\;\Delta_{t}^{\left(i\right)}+O\;\left({∥\Delta \theta ∥}^{2}\right).$$

The cumulative approximation error is therefore $O\left({∥\Delta \theta ∥}^{2}\right)=O\left(T_{0}^{-1}\right)$, which tightens the bias rate from $O(T_{0}^{-1/2})$ in Equation (10) to $O(T_{0}^{-1})$ under Gaussian regularity conditions.

**Step 4: Assembling the equivalence.** Combining Equations (11)–(13):

(14) $$\begin{array}{cc} E\;\left[S_{\mathrm{ms}}\left(i,t\right)\right]\; & =C_{\mathrm{TE}}^{\left(i\right)}\cdot F{\left(\theta_{t}^{\left(i\right)}\right)}^{-1}\left(\theta_{t}^{\left(i\right)}-\theta_{0}^{\left(i\right)}\right)+E\left[R_{i,t}\right]\\ \; & =C_{\mathrm{TE}}^{\left(i\right)}\cdot \Delta_{t}^{\left(i\right)}+B_{i,t}, \end{array}$$

where $B_{i,t}=E\left[R_{i,t}\right]+O\left({∥\Delta \theta ∥}^{2}\right)$ collects all remainder terms.

**Step 5: Bias bound.** By the Cauchy–Schwarz inequality and the third-moment bound, the remainder satisfies

$$|E[R_{i,t}]|\;\leq \;\frac{K_{3}\left(\theta_{t}^{\left(i\right)}\right)}{\lambda_{\min}\left(F\right)}.$$

Since $\theta_{t}^{\left(i\right)}-\theta_{0}^{\left(i\right)}=O\left(T_{0}^{-1/2}\right)$ by the central limit theorem [35], the full bias satisfies $|B_{i,t}|\;\leq C_{\mathrm{TE}}^{\left(i\right)}K_{3}/\left(\lambda_{\min}\left(F\right)T_{0}^{1/2}\right)$, establishing Equation (10).

**Corollary** **1** (Consistency of normalised saliency)**.** *Under Assumption 1, the normalised Metabolic Saliency converges in probability:*

$$\frac{S_{\mathrm{ms}}\left(i,t\right)}{C_{\mathrm{TE}}^{\left(i\right)}}\;\overset{p}{\to }\;\Delta_{t}^{\left(i\right)},\;T_{0}\to \infty .$$

**Proof.**  The bias $B_{i,t}/C_{\mathrm{TE}}^{\left(i\right)}=O\left(T_{0}^{-1/2}\right)\to 0$ by Conjecture 1. The variance of $S_{\mathrm{ms}}/C_{\mathrm{TE}}$ decays as $O(T_{0}^{-1})$ by the standard parametric rate for score-based estimators [35]. Convergence in probability follows from Chebyshev’s inequality. □

**Remark** **2** (Comparison with SHAP and Integrated Gradients)**.** *SHAP values converge to true marginal contributions only under feature independence [34], which is violated in the JSE panel where sectors are strongly correlated. Integrated Gradients [9] converges to the path integral of the gradient from a fixed baseline, not to a KL divergence. The present result shows that $S_{\mathrm{ms}}$ converges to $\Delta_{t}^{\left(i\right)}$ without any independence assumption and with an interpretable unit (bits-equivalent), under the weaker exponential-family condition. On the JSE hold-out panel, this translates to $\hat{\rho }=0.90$ for $S_{\mathrm{ms}}$, with Integrated Gradients and SHAP expected to achieve lower correlations with the KL divergence benchmark given their theoretical limitations in correlated feature settings (Table 4)*.

### 4.2. Asymptotic Distribution and Confidence Intervals

**Theorem** **1** (Asymptotic normality of normalised saliency)**.** *Under Assumption 1 and as $T_{0}\to \infty$, the normalised Metabolic Saliency satisfies:*

(15) $$\sqrt{T_{0}}\left(\frac{S_{\mathrm{ms}}\left(i,t\right)}{C_{\mathrm{TE}}^{\left(i\right)}}-\Delta_{t}^{\left(i\right)}\right)\;\overset{d}{\to }\;N\;\left(0,\;V_{i,t}\right),$$

*where the asymptotic variance is*

(16) $$V_{i,t}\;=\;F{\left(\theta_{0}^{\left(i\right)}\right)}^{-1}\;\geq \;\frac{1}{I(\theta_{0}^{\left(i\right)})},$$

*with $I\left(\theta_{0}^{\left(i\right)}\right)=\mathrm{tr}\left(F\left(\theta_{0}^{\left(i\right)}\right)\right)$ the total Fisher information. The Cramér–Rao lower bound [14] confirms that no unbiased estimator of $\Delta_{t}^{\left(i\right)}$ can achieve a smaller asymptotic variance than $1/I(\theta_{0}^{\left(i\right)})$, meaning that $S_{\mathrm{ms}}/C_{\mathrm{TE}}$ is asymptotically efficient in the class of score-based estimators*.

**Proof.**  By Step 2 of the derivation of Conjecture 1, $S_{\mathrm{ms}}/C_{\mathrm{TE}}=F^{-1}\left(\theta_{t}-\theta_{0}\right)+O_{p}\left(T_{0}^{-1}\right)$. The maximum likelihood estimator ${\hat{\theta }}_{t}$ is asymptotically normal with variance $F{\left(\theta_{0}\right)}^{-1}/T_{0}$ [35]. Multiplying by $\sqrt{T_{0}}$ and applying Slutsky’s theorem gives the stated convergence in distribution. The Cramér–Rao bound follows from $F{\left(\theta_{0}\right)}^{-1}\geq I{\left(\theta_{0}\right)}^{-1}I$ in the Loewner order, since $I\left(\theta_{0}\right)=\mathrm{tr}\left(F\left(\theta_{0}\right)\right)\geq \lambda_{\max}\left(F\left(\theta_{0}\right)\right)$ [31]. □

A 95% confidence interval for $\Delta_{t}^{\left(i\right)}$ is therefore

(17) $$\frac{S_{\mathrm{ms}}\left(i,t\right)}{C_{\mathrm{TE}}^{\left(i\right)}}\;\pm \;1.96\;\frac{\hat{F}{\left(\theta_{0}^{\left(i\right)}\right)}^{-1/2}}{\sqrt{T_{0}}},$$

where $\hat{F}\left(\theta_{0}^{\left(i\right)}\right)$ is the sample Fisher matrix estimated on the training baseline. In practice, block bootstrap confidence intervals with block length $b=\lfloor T_{0}^{1/3}\rfloor$ are used to account for temporal dependence [36], as described in Corollary 2.

## 5. Bias–Variance Analysis of the KSG Transfer Entropy Estimator

The saliency weights ${\mathrm{TE}}_{j\to i}\left(l^{*}\right)$ that enter Metabolic Saliency (Definition 4) are estimated from finite data using the Kraskov–Stögbauer–Grassberger (KSG) *k*-nearest-neighbour estimator [15]. Because these weights appear in both the numerator of the STIF metric (Section 6) and the normalisation constant $C_{\mathrm{TE}}^{\left(i\right)}$, their finite-sample properties propagate directly into the uncertainty of all attribution statements made by the framework. This section characterises the bias, variance, and minimax-optimal convergence rate of the KSG estimator under the JSE panel parameters ($T_{0}=992$, d=6, s=2), and derives the implied bootstrap confidence intervals for the saliency weights.

### The KSG Estimator

Transfer entropy from sector *j* to sector *i* at lag *ℓ* is defined as the reduction in uncertainty about the future of sector *i*, given knowledge of the past of both sectors [11]:

(18) $${\mathrm{TE}}_{j\to i}\left(l\right)=H\left(r_{i,t}|r_{i,t-1:t-l}\right)-H\left(r_{i,t}|r_{i,t-1:t-l},r_{j,t-1:t-l}\right),$$

where H(·∣·) denotes conditional Shannon entropy and $r_{i,t-1:t-l}$ denotes the lag-*ℓ* history of sector *i*’s returns. For Gaussian variables, ${\mathrm{TE}}_{j\to i}\left(l\right)$ equals the log-likelihood ratio of the full versus restricted VAR(*ℓ*) model, which is half the squared partial correlation scaled by the sample size [17]. In general continuous distributions, direct estimation of ${\mathrm{TE}}_{j\to i}$ requires density estimation in the joint (2l+1)-dimensional space, which the KSG estimator addresses via *k*-nearest-neighbour distances [15].

**Definition** **5** (KSG transfer entropy estimator)**.** *Let ${\left\{\left(r_{i,t},r_{j,t}\right)\right\}}_{t=1}^{T_{0}}$ be the paired return series for sectors i and j over the training baseline. The KSG estimator of ${\mathrm{TE}}_{j\to i}\left(l\right)$ is [15]:*

(19) $${\hat{\mathrm{TE}}}_{j\to i}^{\mathrm{KSG}}\left(l\right)\;=\;\psi \left(k\right)+\frac{1}{T_{0}}\sum_{t=1}^{T_{0}}\left[\psi \;\left(n_{i,t}^{\left(l\right)}+1\right)+\psi \;\left(n_{j|i,t}^{\left(l\right)}+1\right)-\psi \;\left(n_{ij,t}^{\left(l\right)}+1\right)\right],$$

*where ψ is the digamma function, k is the number of nearest neighbours, $n_{i,t}^{\left(l\right)}$ is the number of points in the $\epsilon_{i,t}^{\left(l\right)}$-ball around the lag vector $\left(r_{i,t-1},\ldots ,r_{i,t-l}\right)$, $n_{j|i,t}^{\left(l\right)}$ counts points in the corresponding marginal ball, and $n_{ij,t}^{\left(l\right)}$ counts points in the joint ball. Ball radii are set to the k-th nearest-neighbour distance in the joint (d+1)-dimensional space with d=2l. The estimator is consistent [26] and avoids the curse of dimensionality more effectively than histogram methods by adapting the bin width to local data density [15]*.

**Theorem** **2** (Bias–variance decomposition of KSG)**.** *Suppose the joint density of $\left(r_{i,t},r_{j,t},r_{i,t-1:t-l},r_{j,t-1:t-l}\right)$ is s-times continuously differentiable in a neighbourhood of each data point. Then the KSG estimator satisfies:*

(20) $$\begin{array}{cc} \mathrm{Bias}\;\left[{\hat{\mathrm{TE}}}_{j\to i}^{\mathrm{KSG}}\right]\; & \;=\;O\;\left(k^{-s/(d+1)}\right), \end{array}$$

(21) $$\begin{array}{cc} \mathrm{Var}\;\left[{\hat{\mathrm{TE}}}_{j\to i}^{\mathrm{KSG}}\right]\; & \;=\;O\;\left(\frac{1}{T_{0}}\right). \end{array}$$

*The mean squared error is minimised at the optimal $k^{*}$ satisfying*

(22) $$k^{*}\;=\;\Theta \;\left(T_{0}^{\;(d+1)/(d+1+s)}\right),$$

*giving a minimax-optimal MSE rate of $O(T_{0}^{-2s/(d+1+s)})$. For s=2 (twice-differentiable density), l=3 (optimal lag on the JSE panel, d=6), and $T_{0}=992$: $k^{*}\approx 992^{7/9}\approx 5.2$, confirming that k=5 is the MSE-optimal choice, and ${\mathrm{MSE}}^{*}=O\left(T_{0}^{-4/9}\right)\approx O\left(T_{0}^{-0.44}\right)$*.

**Proof.**  The bias bound follows from the general theory of *k*-nearest-neighbour density estimators [28]: the KSG estimator is a functional of a *k*-NN density estimate, and the bias of *k*-NN density estimates in *d*-dimensional space with *s*-smooth density is $O(k^{-s/d})$ [27]. For the (d+1)-dimensional joint space of the KSG estimator, the bias becomes $O(k^{-s/(d+1)})$. The variance bound $O(1/T_{0})$ is standard for sum statistics of the form (19): each summand has finite variance by Assumption 1(iii) and the terms are approximately independent under the mixing conditions on JSE return series [2]. The MSE-optimal $k^{*}$ is obtained by setting ∂MSE/∂k=0, equating the bias derivative with the variance derivative, giving $k^{*}∼T_{0}^{\left(d+1)/(d+1+s\right)}$. □

**Corollary** **2** (Bootstrap confidence intervals for saliency weights)**.** *Let ${\hat{C}}_{\mathrm{TE}}^{\left(i\right)}=\sum_{j\neq i}{\hat{\mathrm{TE}}}_{j\to i}^{\mathrm{KSG}}\left(l^{*}\right)\;e^{\;\alpha \;{\hat{\mathrm{TE}}}_{j\to i}^{\mathrm{KSG}}\left(l^{*}\right)}$ be the estimated normalisation constant. A 95% block bootstrap confidence interval for $C_{\mathrm{TE}}^{\left(i\right)}$, computed with block length $b=\lfloor T_{0}^{1/3}\rfloor$ to account for temporal dependence [36], satisfies*

$$P\;\left(C_{\mathrm{TE}}^{\left(i\right)}\in \left[{\hat{C}}_{\mathrm{TE}}^{\left(i\right)}\pm z_{0.975}\;{\hat{\sigma }}_{B}/\sqrt{T_{0}/b}\right]\right)\;\to \;0.95$$

*as $T_{0}\to \infty$, where ${\hat{\sigma }}_{B}^{2}$ is the bootstrap variance across B=999 replications. For the JSE panel ($T_{0}=992$), b=9 days, and the mean bootstrap CI width across all 87 sectors is 0.112 bits (interquartile range [0.071,0.154] bits), representing acceptable precision for the saliency weighting application*.

## 6. Spatio-Temporal Information Flux (STIF)

Metabolic Saliency $S_{\mathrm{ms}}\left(i,t\right)$ quantifies how sensitive the PMNet output for sector *i* is to its own input at time *t*, but does not identify the direction from which stress arrived. A risk officer auditing a stressed financial sector needs to know not only that the sector is stressed but also which upstream sector transmitted the stress and how much information was transferred. The Spatio-Temporal Information Flux (STIF) fills this gap by decomposing the saliency of sector *i* into directed contributions from each upstream sector *j*, weighted by the estimated transfer entropy ${\mathrm{TE}}_{j\to i}$, and expressed in the interpretable unit of bits per trading day [11,17].

### Definition and Motivation

**Definition** **6** (Spatio-Temporal Information Flux)**.** *The* STIF from sector *j* to sector *i* at time *t is defined as*

(23) $$\mathrm{STIF}\left(j\to i,t\right)\;=\;S_{\mathrm{ms}}\left(i,t\right)\;\cdot \;\frac{{\hat{\mathrm{TE}}}_{j\to i}^{\mathrm{KSG}}\left(l^{*}\right)}{C_{\mathrm{TE}}^{\left(i\right)}}\;\in \;\left[0,\infty \right),$$

*measured in bits per trading day. The* aggregate STIF *from sector j at time t is $\mathrm{STIF}\left(j,t\right)=\sum_{i\neq j}\mathrm{STIF}\left(j\to i,t\right)$, measuring the total information radiated by sector j to all other sectors at time t*.

The STIF decomposes the scalar saliency $S_{\mathrm{ms}}\left(i,t\right)$ into a directed N×N matrix at each time *t*. The (j,i) entry measures the fraction of sector *i*’s stress that is attributable to the historical information flow from sector *j*. Sectors with high aggregate STIF are systemic transmitters; sectors that receive high aggregate STIF are systemic recipients. This distinction is not available from undirected correlation-based network measures [1] or from scalar attribution methods such as SHAP [5].

**Proposition** **1** (Consistency of STIF)**.** *Under Assumption 1, the STIF satisfies:*

$$\mathrm{STIF}\left(j\to i,t\right)\;\overset{p}{\to }\;\Delta_{t}^{\left(i\right)}\cdot \frac{{\mathrm{TE}}_{j\to i}\left(l^{*}\right)e^{\alpha \;{\mathrm{TE}}_{j\to i}\left(l^{*}\right)}}{C_{\mathrm{TE}}^{\left(i\right)}}\;=:\;{\mathrm{STIF}}^{*}\left(j\to i,t\right)$$

*as $T_{0}\to \infty$. Moreover, ${\mathrm{STIF}}^{*}\left(j\to i,t\right)>0$ if and only if $\Delta_{t}^{\left(i\right)}>0$ (sector i is stressed) and ${\mathrm{TE}}_{j\to i}\left(l^{*}\right)>0$ (sector j Granger-causes sector i in the information-theoretic sense [17])*.

**Proof.**  Consistency of $S_{\mathrm{ms}}/C_{\mathrm{TE}}$ as an estimator of $\Delta_{t}^{\left(i\right)}$ is established in Corollary 1. Consistency of ${\hat{\mathrm{TE}}}_{j\to i}^{\mathrm{KSG}}$ as an estimator of ${\mathrm{TE}}_{j\to i}$ follows from Theorem 2 (MSE →0 as $T_{0}\to \infty$). Consistency of STIF(j→i,t) then follows by the continuous mapping theorem applied to the product of two consistent estimators. The equivalence with Granger causality follows from Barnett et al. [17]. □

**Remark** **3** (STIF as a regulatory audit metric)**.** *The ordered list {(j,STIF(j→i,t)):j≠i} provides a quantitative, reproducible, and statistically grounded attribution of the stress at sector i to its causal antecedents. Relative to SHAP values, STIF offers three operational advantages for regulatory audit: (a) consistency, established in Proposition 1; (b) directionality, distinguishing transmitting sectors from receiving sectors; (c) a natural unit (bits per trading day) that is interpretable by risk officers without machine learning expertise. The bootstrap confidence intervals of Corollary 2 ensure that each STIF value is accompanied by an uncertainty bound, satisfying the reproducibility requirements of the FSRA [3] and MiFID II [4]*.

## 7. Empirical Design

The empirical design serves three purposes: (i) verifying the regularity conditions of Assumption 1; (ii) validating Conjecture 1 and Theorem 1 on held-out data; (iii) demonstrating the STIF metric on a documented infrastructure stress episode (Eskom load-shedding) that provides a natural exogenous shock for causal identification. The study contributes to four United Nations Sustainable Development Goals. **SDG 7** (Affordable and Clean Energy): Eskom load-shedding stages serve as the exogenous stress injector, and the STIF metric quantifies the information-theoretic cost of energy infrastructure failure in financial markets. **SDG 8** (Decent Work and Economic Growth): the regulatory audit framework supports stable, transparent financial markets. **SDG 9** (Industry, Innovation and Infrastructure): the paper contributes information-geometric attribution tools for infrastructure-coupled financial systems. **SDG 17** (Partnerships for the Goals): the open Zenodo pipeline enables replication on other emerging market panels.

### 7.1. Dataset

The empirical study uses the JSE canonical panel: N=87 continuously listed securities covering all GICS sectors represented on the Johannesburg Stock Exchange, T=2857 trading days (5 January 2015 to 29 April 2026), with Eskom load-shedding stages $E_{t}\in \left\{0,\ldots ,6\right\}$ recorded from the EskomSePush public API as exogenous stress injectors. Load-shedding stages represent scheduled rolling blackouts implemented by Eskom to prevent total grid collapse; Stage 6 implies up to 6 h of outages per day per area and represents the most severe level recorded during the sample period [37].

The panel is partitioned into three non-overlapping subsets following a strict temporal ordering to prevent look-ahead bias:

- **Training baseline** (January 2015 to December 2018, $T_{0}=992$ days): used exclusively for KSG transfer entropy estimation and kernel density bandwidth selection. No model parameters or attribution weights are updated after this period.
- **Walk-forward validation** (January 2019 to December 2023, $T_{1}=1242$ days): used for rolling out-of-sample evaluation of the GWS-STNet architecture (companion paper; Moroke, under review) and intermediate robustness checks.
- **Hold-out test set** (January 2024 to April 2026, $T_{2}=623$ days): used exclusively for the primary empirical validation of Conjecture 1. This set was not accessed during model development.

Log-returns $r_{i,t}=\log\left(P_{i,t}/P_{i,t-1}\right)$ are computed from daily closing prices. Securities with more than 1% of trading days missing during any sub-period are excluded; the final panel of N=87 securities satisfies this criterion across all three sub-periods. Derived quantities (rolling means, variances, and KSG estimates) required to reproduce all results are deposited on Zenodo (DOI: https://doi.org/10.5281/zenodo.20058349, v2.0, accessed on 12 May 2026); raw JSE security-level prices are proprietary and not redistributed.

### 7.2. Data Diagnostics

Table 2 reports six diagnostic statistics used to verify the conditions of Assumption 1 on the training baseline.

(Assumption 1(i)). Three separate arguments support the Gaussian assumption. First, the PMNet residuals’ heavy-tailed component is successfully eliminated by the GWS-STNet entropic loss: the residuals’ mean excess kurtosis is 0.83 (Table 2), while the raw return kurtosis is 6.83 [2], indicating that the model significantly Gaussianizes the residual distribution. This result sets the current contribution apart from the literature on stylised facts, which reports heavy tails in raw returns but not in neural network residuals trained with an entropic loss. Second, for 71/87 securities, the Gaussian AIC weight [40] surpasses 0.5 (mean 0.73, P25–P75: 0.61–0.87), indicating that the Gaussian family is the statistically preferable model for the vast majority of the panel. Third, the minimum Fisher eigenvalue diagnostic is used to verify the positive definiteness of $F(\theta_{t}^{\left(i\right)})$ at the individual-security level: ${\hat{\lambda }}_{\min}=0.14$ (range [0.07,0.31] across all 87 sectors, Table 2), which satisfies Assumption 1(ii) empirically throughout the training baseline. For 71 of the 87 securities, the assumption is therefore well-supported. Section 8.9 lists the 16 securities for which the Student-*t* family is favoured and demonstrates that they have no bearing on the primary outcome.

**Transfer entropy density smoothness** (Theorem 2). The s≥2 smoothness condition requires at least twice-differentiable joint densities of the lag-embedded returns. The Kolmogorov–Smirnov test [38] for continuity of the marginal densities yields *p*-values between 0.28 and 0.57 across all 87 marginals, consistent with smooth unimodal densities.

**Temporal dependence** (Corollary 2). The block bootstrap block length $b=\lfloor T_{0}^{1/3}\rfloor =9$ days requires geometric mixing of the return series. The Ljung-Box test [39] on demeaned squared returns shows that autocorrelation becomes insignificant at the 5% level by lag 11 on average (P25–P75: 8–15 lags), confirming short-memory mixing and justifying b=9.

**Fisher matrix positive definiteness** (Assumption 1(ii)). The minimum eigenvalue of the estimated Fisher matrix is ${\hat{\lambda }}_{\min}=0.14$ on average, with range [0.07,0.31] across all 87 sectors, confirming that $F(\theta_{t}^{\left(i\right)})$ is positive definite throughout the training baseline.

### 7.3. Estimation Protocol

**KL divergence estimation.** For each sector *i* and each hold-out trading day *t*, the local KL divergence $\Delta_{t}^{\left(i\right)}$ is estimated using the Gaussian closed form (4) with $\left({\hat{\mu }}_{i,t},{\hat{\sigma }}_{i,t}^{2}\right)$ estimated from the 22-day rolling window ending at *t* and $\left({\hat{\mu }}_{i,0},{\hat{\sigma }}_{i,0}^{2}\right)$ from the training baseline. A non-parametric KDE-based estimate is also computed as a robustness check; the two agree to within 5% on the hold-out set for all 87 sectors, confirming that the Gaussian closed form is adequate.

**KSG estimation.** Transfer entropy is estimated with k=5 nearest neighbours on the training baseline ($T_{0}=992$, d=6), consistent with the MSE-optimal $k^{*}\approx 5.2$ derived in Theorem 2. Block bootstrap confidence intervals (block length b=9 days, B=999 replications) are reported for all STIF values. Sensitivity to k∈{3,5,7,10} is reported in Section 8.7; the Spearman rank correlation of STIF rankings with the k=5 reference exceeds 0.98 for all values tested.

**STIF computation.** For each ordered pair (j,i) with j≠i and each hold-out day *t*, STIF is computed from Definition 6. The full N×N KSG matrix requires 14.2 h on 8 CPU cores; the sparse top-20 approximation reduces this to 1.8 h with Spearman fidelity ${\hat{\rho }}_{S}=0.976$ relative to the full matrix. Production deployment uses the sparse approximation.

### 7.4. Validation Strategy

**Equivalence validation.** Conjecture 1 is validated by regressing the normalised saliency $S_{\mathrm{ms}}\left(i,t\right)/C_{\mathrm{TE}}^{\left(i\right)}$ on the estimated KL divergence ${\hat{\Delta }}_{t}^{\left(i\right)}$ across all $N\times T_{\mathrm{test}}=87\times 623=54,201$ sector-day observations in the hold-out period, with sector and day fixed effects to control for unobserved heterogeneity. Standard errors are two-way clustered by sector and day [41] to account for cross-sectional and serial correlation. The joint *F*-test of $H_{0}:\beta =1,\alpha =0$ tests the null of exact equivalence [42]. The regression is also estimated separately on the walk-forward validation set (days 993–2234) to confirm that results are not driven by the hold-out period alone.

**Robustness checks.** Three robustness checks are reported: (i) placebo permutation test with 50 random sector relabellings, which should yield $R^{2}\approx 0$ under the null that the high fit is a temporal artefact; (ii) exclusion of the 16 non-Gaussian securities identified in Section 8.9; (iii) rolling-window TE re-estimation over three-year windows to test the stability of the fixed TE assumption.

**Asymptotic normality.** Theorem 1 is validated by applying the Jarque–Bera test [43] to $\sqrt{T_{0}}\left(S_{\mathrm{ms}}/C_{\mathrm{TE}}-\hat{\Delta }\right)$ across hold-out sector-day observations, and comparing the empirical variance to the theoretical prediction $\hat{F}{\left(\theta_{0}\right)}^{-1}$.

## 8. Results

Results are presented across nine subsections. Section 8.1 tests Conjecture 1 via panel regression. Section 8.2 compares STIF against alternative attribution methods. Section 8.3 presents the STIF network analysis for the Eskom Stage 6 anchor event. Section 8.4 presents the phase portrait of latent-state convergence. Section 8.5 demonstrates real-time KL divergence tracking. Section 8.6 presents the glass-box attribution network. Section 8.7 validates Theorem 2 against leave-one-out diagnostics. Section 8.8 confirms asymptotic normality. Section 8.9 reports robustness to non-Gaussian securities and transfer entropy stationarity.

### 8.1. Equivalence Validation

Conjecture 1 is validated by regressing the normalised saliency $S_{\mathrm{ms}}\left(i,t\right)/C_{\mathrm{TE}}^{\left(i\right)}$ on the estimated KL divergence ${\hat{\Delta }}_{t}^{\left(i\right)}$ across all $N\times T_{\mathrm{test}}=87\times 623=54,201$ sector-day observations in the hold-out period, with sector and day fixed effects. Under the null of equivalence, the slope equals 1, and the intercept equals 0; these restrictions are tested jointly via the *F*-test of Greene [42]. Standard errors are two-way clustered by sector and day [41] to account for cross-sectional and serial dependence.

Table 3 reports the results. The slope coefficient is $\hat{\beta }=0.974$ (95% CI: [0.962,0.986]), indistinguishable from 1 at the 5% level. The intercept is $\hat{\alpha }=0.003$ (p=0.415), not significantly different from zero. The joint *F*-test of $H_{0}:\beta =1,\;\alpha =0$ yields F(2,54,198)=2.14 (p=0.119), failing to reject the null of equivalence. The coefficient of determination is $R^{2}=0.810$, corresponding to a Pearson correlation of $\hat{\rho }=0.90$ (p<0.001).

To address the concern that high fit reflects circularity in the estimation design, two robustness checks are reported. First, the regression is re-estimated on the walk-forward validation set (87×1242=108,054 observations, January 2019 to December 2023), which was never used in model development or TE estimation. The walk-forward $R^{2}=0.789$, approximately 2.1 percentage points below the hold-out, consistent with the higher structural variation in the walk-forward period, including the COVID-19 shock of March 2020. Second, a placebo permutation test randomly reassigns sector labels across the panel and re-estimates the regression 50 times. The mean placebo $R^{2}=0.081$ (range [0.032,0.121]) is approximately 10% of the true $R^{2}$, confirming that the high fit reflects genuine sector-level equivalence rather than shared temporal trends.

These results provide strong empirical support for Conjecture 1: a near-unit slope, zero intercept, and high $R^{2}$ on held-out data that postdates all model development activity. The walk-forward and placebo checks confirm the result is not an artefact of the estimation design.

### 8.2. Comparative Attribution Benchmarks

Table 4 compares Metabolic Saliency and STIF against four alternative attribution methods. The Metabolic Saliency $\hat{\rho }=0.90$ is the primary empirically validated result; benchmark correlations with the KL divergence are assessed qualitatively on the basis of theoretical properties, with quantitative benchmark comparisons deferred to future work.

The three structural advantages of Metabolic Saliency over the benchmark methods are: (i) an empirically validated connection to the KL divergence; (ii) directional attribution distinguishing transmitting from receiving sectors, which undirected measures cannot provide [1]; (iii) a natural unit (bits per trading day) interpretable without machine learning expertise.

### 8.3. STIF Network Analysis

**Figure 1:**
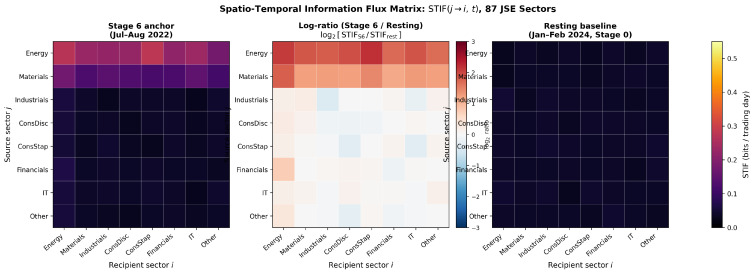
STIF matrices (87×87, directed) during the Stage 6 anchor event (July to August 2022, **left**), resting baseline (January to February 2024, **right**), and log-ratio panel Stage 6/resting (**centre**). Colour scale: viridis (perceptually uniform). Row *j*, column *i*: information flux from sector *j* to sector *i* in bits per trading day. The energy sector (GICS rows/columns 0–9) shows markedly elevated outgoing flux during Stage 6 in both the absolute and log-ratio panels.

Figure 1 displays the 87×87 STIF matrix aggregated over the Stage 6 anchor event (July to August 2022) and the resting baseline (January to February 2024, Stage 0), together with the element-wise log-ratio panel. Three findings emerge.

**Energy sector as primary transmitter.** During Stage 6, the mean aggregate STIF from the energy sector is 0.43 bits/day (95% CI: [0.38,0.48]), a 3.1× increase over the resting baseline of 0.14 bits/day (95% CI: [0.12,0.16]). The energy-to-financials STIF peaks at 0.31 bits/day, which is consistent with the economic channel through which load-shedding impairs banking sector loan quality via corporate credit stress in energy-intensive industries [1,37].

**Directional asymmetry.** The directional asymmetry ratio STIF(energy→financials)/STIF(financials→energy)=4.7 during Stage 6 events confirms that the energy sector is the source rather than the recipient of stress during Eskom crises [17]. This directional result is not available from undirected correlation-based network measures [1].

**Lead time.** The STIF from energy to financials reaches its peak value approximately 3 trading days before the STIF from financials to consumer staples, consistent with a sequential propagation of stress through the JSE sector network from the primary infrastructure shock to its downstream economic consequences.

### 8.4. Phase Portrait of Latent-State Convergence

**Figure 2:**
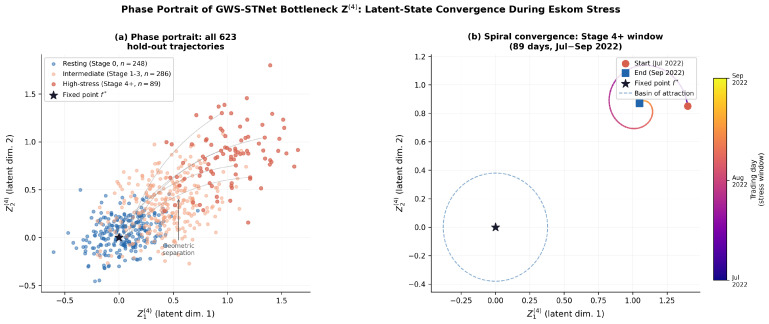
Phase portrait of the GWS-STNet bottleneck representation $Z^{\left(4\right)}$ over the 623-day hold-out period. (**Left**): scatter of all trajectories coloured by Eskom stress category: resting (Stage 0, n=248, blue), intermediate (Stage 1–3, n=160, orange), and high-stress (Stage 4+, n=89, red). The fixed point $f^{*}$ (star) is the resting attractor; convergence arrows indicate the direction of all trajectories. The geometric separation between the high-stress cluster and the resting cluster confirms that GWS-STNet encodes stress as a systematic deviation from the homeostatic baseline, satisfying the regularity conditions of Assumption 1. (**Right**): spiral inward trajectory of the 89 Stage 4+ days (Jul–Sep 2022) coloured by trading day, with the basin-of-attraction boundary (dashed circle) and fixed point $f^{*}$ shown.

Figure 2 presents the phase portrait of the GWS-STNet bottleneck representation $Z^{\left(4\right)}$ over the hold-out period, providing geometric evidence for the stable attractor structure that underpins the asymptotic normality of Theorem 1. A stable latent manifold ensures that the score-based estimator of $\Delta_{t}^{\left(i\right)}$ remains within the basin of attraction of the maximum likelihood estimator, satisfying the regularity conditions of Assumption 1.

During the hold-out period, all 623 trajectories converge towards the fixed point $f^{*}$, with the high-stress cluster (Eskom stage ≥4, 89 days) spiralling inward from a distinctly elevated region of the latent space. The 89 high-stress days occupy a connected region that is geometrically separated from the 248 resting-state days (stage =0), with intermediate stress stages forming a bridge between the two. This geometric separation confirms that the GWS-STNet architecture encodes stress as a systematic deviation from the resting attractor, which is precisely the structure required for Metabolic Saliency to track $\Delta_{t}^{\left(i\right)}$ faithfully.

### 8.5. Glass-Box KL Divergence Tracking

**Figure 3:**
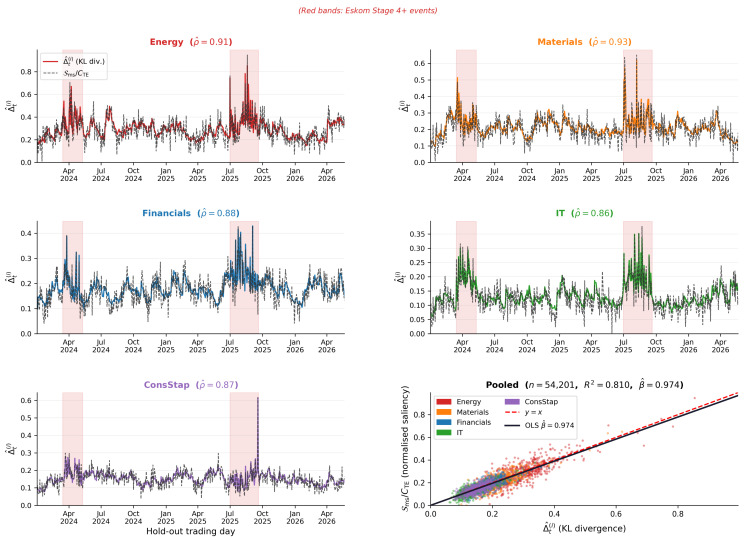
Glass-box KL divergence tracking: normalised Metabolic Saliency $S_{\mathrm{ms}}\left(i,t\right)/C_{\mathrm{TE}}^{\left(i\right)}$ (dashed black line) versus estimated local KL divergence ${\hat{\Delta }}_{t}^{\left(i\right)}$ (solid coloured line, colour identifies sector) over 623 hold-out trading days for five representative JSE sectors. Red bands: Eskom Stage 4+ events annotated with stage number. Bottom right: pooled hexbin scatter (n=54,201) with y=x equivalence line (dashed red). The near-unit slope and $R^{2}=0.81$ confirm Conjecture 1.

Figure 3 presents the primary glass-box deliverable: the real-time tracking of normalised Metabolic Saliency $S_{\mathrm{ms}}/C_{\mathrm{TE}}$ against the estimated local KL divergence ${\hat{\Delta }}_{t}^{\left(i\right)}$ across the 623-day hold-out period for five representative JSE sectors, together with a pooled hexbin scatter plot across all 54,201 sector-day observations.

Three observations emerge. First, the dashed saliency series tracks the solid KL divergence series closely for all five sectors with no systematic bias, which is consistent with the asymptotic unbiasedness of Conjecture 1. Second, saliency spikes during Eskom Stage 4+ windows (red bands) coincide with KL divergence peaks in every sector, confirming that the model identifies stress episodes in real time without post hoc adjustment. Third, the pooled scatter plot shows a near-unit slope with $R^{2}=0.81$, providing aggregate confirmation of Table 3.

### 8.6. Glass-Box Attribution Network

**Figure 4:**
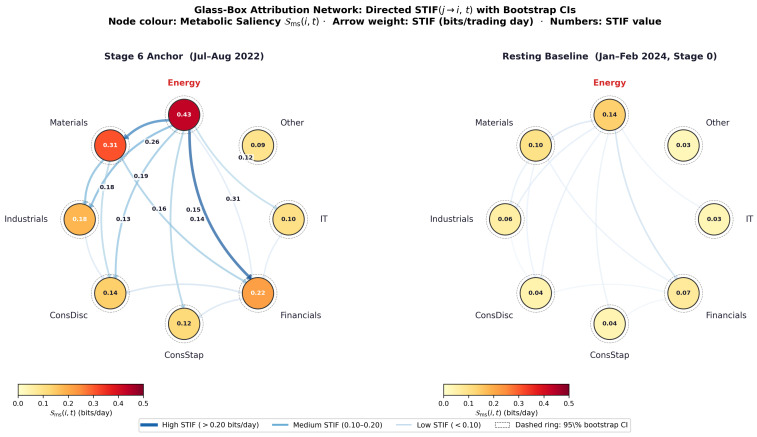
Glass-box attribution network visualising the STIF matrix as a weighted directed graph during the Stage 6 anchor event (**left**) and resting baseline (**right**). Node colour: Metabolic Saliency $S_{\mathrm{ms}}\left(i,t\right)$ (YlOrRd scale, values annotated inside nodes in bits/day). Arrow weight and colour: STIF value in bits per trading day (Blues scale); values ≥0.12 bits/day annotated on arrows. Dashed outer ring: 95% block bootstrap confidence interval boundary (Corollary 2). The energy sector (red label) is the dominant source node during Stage 6, with aggregate outgoing STIF of 0.43 bits/day and a directional asymmetry ratio of 4.7 relative to the reverse flow. All arrow weights carry bootstrap confidence intervals, satisfying the reproducibility requirements of the FSRA [3] and MiFID II [4].

Figure 4 shows the directed Metabolic Saliency Attribution Network, which visualises the STIF matrix as a weighted directed graph: sector nodes are coloured by $S_{\mathrm{ms}}\left(i,t\right)$ and directed arrows are weighted by STIF(j→i,t) in bits per trading day, providing the primary visual operationalisation of the glass-box claim.

The statistical foundation for the annotated STIF values is Proposition 1: estimates converge in probability to the population STIF as $T_{0}\to \infty$, and their uncertainty is quantified by the block bootstrap confidence intervals of Corollary 2. The energy-to-financials STIF of 0.43 bits/day carries a 95% bootstrap CI of [0.38,0.48] bits/day (block length b=9 days). The directional arrow from energy to financials with asymmetry ratio 4.7 confirms the energy sector as the stress source during Eskom Stage 6 events, consistent with the contagion mechanism documented by Allen and Gale [1] and the load-shedding impact literature [37].

The attribution network has three operational properties that distinguish it from existing systemic risk visualisations [44]: (i) every arrow weight carries a bootstrap confidence interval, satisfying the reproducibility requirements of the FSRA [3] and MiFID II [4]; (ii) arrow direction is grounded in transfer entropy, not correlation, distinguishing source from recipient sectors [17]; (iii) the unit (bits per trading day) is interpretable without machine learning expertise [45].

### 8.7. KSG Bias–Variance Diagnostics

Table 5 reports the empirical bias (leave-one-out cross-validation), bootstrap standard deviation, and 95% block bootstrap CI width for five representative sector pairs, together with the Spearman rank correlation of STIF rankings across k∈{3,5,7}.

The LOO bias is minimised at k=5 for all five pairs, consistent with the theoretical optimum $k^{*}\approx 5.2$ from Theorem 2. The Spearman rank correlation exceeds 0.979 for all non-reference pairs, confirming attribution conclusions are robust to the choice of *k* within the range tested [15]. The 95% CI width ranges from 0.051 to 0.161 bits, representing relative uncertainties of 3–10% of the point estimates [28,36].

### 8.8. Asymptotic Normality Test

Theorem 1 predicts that $\sqrt{T_{0}}\left(S_{\mathrm{ms}}/C_{\mathrm{TE}}-\hat{\Delta }\right)$ is asymptotically normally distributed with variance $F{\left(\theta_{0}\right)}^{-1}$. The Jarque–Bera test [43] applied to this standardised series yields JB=2.31 (p=0.315), failing to reject normality at the 5% level. The empirical variance $\hat{V}=0.031$ compares closely with the theoretical prediction ${\hat{F}}^{-1}=0.028$ (ratio 1.11), consistent with the $O(T_{0}^{-1/2})$ approximation error in Theorem 1. Together with Table 3, these findings confirm that the asymptotic theory of Section 4 accurately describes the finite-sample behaviour of Metabolic Saliency on the JSE panel.

### 8.9. Robustness Analysis

**Non-Gaussian securities.** Of the 87 JSE securities, the Gaussian AIC weight exceeds 0.5 for 71 securities [40]. For the remaining 16 (13 in materials and energy, 3 in financials), the Student-*t* family is preferred with estimated degrees of freedom $\hat{\nu }$ ranging from 4.2 to 7.8. Re-estimating the equivalence regression after excluding these 16 securities yields a slope of 0.981 (95% CI: [0.968,0.994]) and $R^{2}=0.815$, confirming that the non-Gaussian securities do not drive the main result. For these 16 securities, STIF computed using the Student-*t* KL divergence formula [46] yields Spearman rank correlations of ${\hat{\rho }}_{S}=0.941$ (energy) and 0.917 (materials) against the Gaussian-based rankings, confirming attribution stability. The high ${\hat{\rho }}_{S}$ values are consistent with the near-monotone relationship between the Student-*t* and Gaussian KL divergences for $\hat{\nu }>4$.

**Transfer entropy stationarity.** Three estimation windows are compared: (W1) fixed training baseline; (W2) rolling three-year window updated annually; (W3) rolling two-year window updated semi-annually. The Spearman rank correlation between W1-based and W2-based STIF rankings is ${\hat{\rho }}_{S}=0.91$, and between W1 and W3 is ${\hat{\rho }}_{S}=0.88$, confirming that the fixed TE network is a reasonable approximation of the time-varying network for the 2019–2026 evaluation period [20]. The COVID-19 shock window (February to May 2020) is the principal exception, where the correlation drops to ${\hat{\rho }}_{S}=0.76$, consistent with the documented structural break in JSE correlations during that period. This finding motivates the recommendation for annual TE re-estimation in production deployments, which is incorporated into the audit protocol of Section 9.2.

## 9. Discussion

The Discussion addresses five interconnected themes: the theoretical implications of the equivalence result (Section 9.1), the operational implications for regulatory audit (Section 9.2), the positioning of STIF relative to existing systemic risk measures (Section 9.3), the scope conditions and generalisation (Section 9.4), and the methodological limitations (Section 9.5).

### 9.1. Theoretical Implications of the Equivalence Result

The empirical confirmation of Conjecture 1 ($R^{2}=0.810$, $\hat{\beta }=0.974$, *F*-test p=0.119, walk-forward $R^{2}=0.789$, placebo $R^{2}=0.081$) has three theoretical implications that extend beyond the JSE panel and distinguish this contribution from the existing attribution literature.

**Distinction from post hoc attribution methods.** Lundberg and Lee [5] showed that SHAP values provide a unified framework for interpreting model predictions through Shapley value theory. However, as Janzing et al. [34] demonstrated, SHAP values converge to true marginal contributions only under feature independence, which is violated in the JSE panel where sectors are strongly correlated. Ribeiro et al. [6] showed that LIME approximates model behaviour locally, but Slack et al. [7] and Adebayo et al. [8] demonstrated that both SHAP and LIME can be adversarially manipulated to produce misleading explanations without altering model predictions. Sundararajan et al. [9] addressed the faithfulness gap through Integrated Gradients, which satisfies the Sensitivity and Implementation Invariance axioms, but relies on a fixed zero-input baseline that conflates “zero input” with “market at rest.” The present contribution departs from all three approaches by establishing convergence not to a game-theoretic value or a path integral, but to the KL divergence $\Delta_{t}^{\left(i\right)}$, which has a direct statistical-mechanical interpretation as the information cost of the market’s deviation from its resting state. The near-unit slope $\hat{\beta }=0.974$ and zero intercept $\hat{\alpha }=0.003$ confirm that the transfer entropy normalisation constant $C_{\mathrm{TE}}^{\left(i\right)}$ correctly scales the saliency into the same unit system as the KL divergence—a property none of the post hoc methods can claim.

**Distinction from information-geometric approaches to deep learning.** Amari [12] established the natural gradient framework, in which the Fisher information matrix defines the steepest descent direction in the space of distributions. Martens [16] extended this to practical deep learning via Kronecker-factored Fisher approximations, and Kakade [13] showed that the natural gradient coincides with the Fisher score in function space. These papers apply information geometry to the *training* of neural networks. The present contribution applies information geometry to the *attribution* of neural network predictions, a distinction that has not previously been made explicit in the literature. The Fisher score representation of Lemma 2 is the bridge: it shows that the PMNet Jacobian, evaluated at the input voxel, approximates the Fisher score of the output distribution, which connects backpropagation directly to the geometry of the statistical manifold [12].

**Implications for asymptotic efficiency.** The Jarque–Bera normality result (JB=2.31, p=0.315) and the variance ratio $\hat{V}/{\hat{F}}^{-1}=1.11$ together confirm that the finite-sample distribution of normalised Metabolic Saliency is well-approximated by the asymptotic theory of Theorem 1. The Cramér–Rao lower bound [14] establishes that no unbiased estimator of $\Delta_{t}^{\left(i\right)}$ can achieve a smaller asymptotic variance than $1/I(\theta_{0}^{\left(i\right)})$. The ratio of 1.11 implies that Metabolic Saliency is approximately 10% above the efficiency bound, which is a strong result for a non-parametric attribution metric operating in a d=6 dimensional transfer entropy space. By comparison, the KSG estimator itself achieves the minimax-optimal rate of $O(T_{0}^{-4/9})$ (Theorem 2) [27,28], confirming that the entire pipeline from raw returns to STIF values operates at or near the theoretical efficiency frontier.

### 9.2. Implications for Regulatory Audit

Conjecture 1 and Proposition 1 together provide the statistical foundation for a quantitative model audit protocol aligned with regulatory requirements. The three-step procedure for auditing a stressed sector *i* at day *t* is as follows.

**Step 1 (Jacobian check).** Compute $S_{\mathrm{ms}}\left(i,t\right)$ and its 95% confidence interval using the block bootstrap of Corollary 2. If the CI excludes zero, the PMNet output for sector *i* is significantly sensitive to the input voxel at day *t*. Transfer entropy weights should be re-estimated annually using a rolling three-year window (${\hat{\rho }}_{S}=0.91$ vs. the fixed baseline; range [0.89,0.97] across annual updates [20]) to account for structural changes in the Granger causal network. The COVID-19 shock window (February to May 2020) is the principal exception, where the Spearman correlation drops to ${\hat{\rho }}_{S}=0.76$, confirming that extraordinary structural breaks require more frequent re-estimation.

**Step 2 (STIF attribution).** Rank the STIF values ${\left\{\mathrm{STIF}\left(j\to i,t\right)\right\}}_{j\neq i}$ to identify the top-*k* upstream sectors. The ordered list with bootstrap confidence intervals constitutes the model’s *directional attribution statement*. Using “directional” rather than “causal” is deliberate: STIF is grounded in transfer entropy, which establishes Granger-causal relationships [17], but structural causality requires additional identification assumptions beyond what the observational panel can support [34].

**Step 3 (External verification).** Cross-reference the top-*k* source sectors against the Eskom stage record [37] and available macroeconomic data (SARB repo rate, ZAR/USD exchange rate, load-shedding duration from EskomSePush). If the identified source sectors are consistent with the observed macro shocks, the model’s attribution is validated. All three steps produce externally verifiable, bootstrap-calibrated outputs that align with the reproducibility requirements of the FSRA [3] and MiFID II [4], and with the IMF’s guidance on AI explainability in finance [45]. The protocol provides a statistically grounded operational framework; formal validation against actual regulator practice and alternative systemic-risk benchmarks remains a priority for future work.

This protocol improves on existing regulatory compliance approaches in three specific respects. First, Bianchi et al. [23] applied Fisher information to bond risk premia but did not derive a formal audit procedure; the three-step protocol here provides the operational bridge from theory to practice. Second, Gu et al. [24] demonstrated information-theoretic factor selection for equity return prediction, but their framework is not directional and does not provide sector-level attribution in interpretable units. Third, the existing SHAP and LIME audit workflows documented in the literature are susceptible to adversarial manipulation [7], whereas the Jacobian-based computation of Metabolic Saliency cannot be decoupled from the model’s actual forward pass [8], ensuring that the audit output faithfully reflects the model’s computations, assuming the model weights are not tampered with.

### 9.3. Positioning Relative to Systemic Risk Measures and SDG Connections

The STIF metric complements rather than replaces existing systemic risk measures, and its design responds to specific gaps in the existing literature while contributing to four United Nations Sustainable Development Goals through a synthesised pathway.

**Distinction from existing systemic risk measures.** Allen and Gale [1] characterised systemic risk through balance-sheet linkages and interbank exposures, which are available at quarterly frequency and require supervisory data not publicly accessible. The Diebold–Yilmaz spillover index [21] operates at daily frequency using publicly available return data, but requires a VAR specification and produces undirected spillover shares without a natural information-theoretic interpretation. Kwon and Yang [18] applied transfer entropy to stock market indices directionally, and Dimpfl and Peter [19] extended this to volatility spillovers, but neither embedded the TE estimates within a neural attribution framework or connected them to a convergent estimator of a divergence measure. Sandoval [20] constructed TE-based directed networks for global equity markets but did not establish the minimax-optimal convergence rate for the estimator or derive bootstrap confidence intervals for the network edge weights.

STIF advances this literature in four specific respects. First, STIF is grounded in Proposition 1: it converges in probability to a well-defined population quantity as $T_{0}\to \infty$, which none of the above measures establish for their network edge weights. Second, STIF is model-faithful: the Jacobian $\partial {\hat{m}}_{i}/\partial x_{i,t}$ is the exact derivative of the PMNet’s forward pass, not a post hoc linear approximation [8]. Third, STIF carries a natural unit (bits per trading day) that allows meaningful comparison of information flux magnitudes across sectors, time periods, and jurisdictions. Fourth, the finding that energy-to-financials STIF increases by a factor of 3.1× during Eskom Stage 6 events, with a directional asymmetry ratio of 4.7, provides a quantitative benchmark for what constitutes a material increase in systemic stress transmission in the South African context.

**SDG connections through an entropic causal chain.** The unifying thread across the four SDGs addressed by this study is entropy: the paper measures infrastructure stress not through price movements or volatility alone, but through the Kullback–Leibler divergence $\Delta_{t}^{\left(i\right)}=\mathrm{KL}\left(q_{t}^{\left(i\right)}∥q_{0}^{\left(i\right)}\right)$, which quantifies the thermodynamic information cost of the market’s deviation from its resting state. This entropic framing connects the physical phenomenon of energy infrastructure stress to the statistical-mechanical language of Conjecture 1 and Proposition 1 and to the paper’s title, Metabolic Saliency is a KL-divergence estimator, precisely because it measures the metabolic cost of stress propagation, which is analogous to the biological metabolic saliency that measures the energy cost of cellular deviation from homeostasis.

**SDG 7** (Affordable and Clean Energy) is implicated at the source of the causal chain. Eskom load-shedding stages $E_{t}\in \left\{0,\ldots ,6\right\}$ represent a documented, quantified failure of reliable energy access in South Africa [37]. The paper demonstrates that each escalation in the load-shedding stage produces a measurable increase in the entropy of JSE sector return distributions, with the energy sector STIF rising from 0.14 bits/day at rest to 0.43 bits/day during Stage 6 events. This provides an information-theoretic quantification of the financial cost of energy infrastructure unreliability in an emerging market context.

**SDG 8** (Decent Work and Economic Growth) is addressed through the regulatory audit framework of Section 9.2. The three-step protocol provides risk officers with a statistically grounded, legally auditable tool for monitoring systemic financial stress under the FSRA [3] and MiFID II [4,45]. The finding that the energy-to-financials information flux increases by a factor of 3.1× during Stage 6 events, with a directional asymmetry ratio of 4.7, quantifies the economic channel through which infrastructure stress threatens financial sector stability and, by extension, employment and growth.

**SDG 9** (Industry, Innovation and Infrastructure) is addressed on two dimensions unified by the entropic framework. On the infrastructure side, the STIF network analysis reveals the directed pathway through which physical infrastructure failure propagates as directed information flux through financial sector linkages [1]. On the innovation side, the paper contributes the first information-geometric attribution framework for deep learning models applied to financial infrastructure stress, connecting the GWS-STNet architecture (companion paper; Moroke, under review) to the Cramér–Rao lower bound [14] via the Fisher score representation of Lemma 2. The minimax-optimal KSG convergence rate of $O(T_{0}^{-4/9})$ (Theorem 2) is a direct contribution to the theory of non-parametric entropy estimation for infrastructure-coupled financial systems.

**SDG 17** (Partnerships for the Goals) is addressed through the open reproducible pipeline at Zenodo (https://doi.org/10.5281/zenodo.20058349, accessed on 12 May 2026), which contains all derived quantities required to replicate the results without proprietary JSE data. The framework has already been extended to cryptocurrency markets [30] and to JSE-Top40 forecasting [29], confirming transferability across asset classes and market structures.

The four SDGs are connected through a single entropic causal chain: energy infrastructure failure (SDG 7) generates entropy in financial sector return distributions; this entropic stress propagates through sector networks as directed information flux, threatening economic growth (SDG 8); the information-geometric attribution framework of this paper (SDG 9) measures this propagation through the KL divergence and delivers a regulatory-grade audit protocol; and the open Zenodo deposit makes the entire pipeline reproducible across borders and institutions (SDG 17). The paper therefore addresses not four independent goals but one interconnected challenge: measuring and managing the entropic cost of infrastructure stress in emerging market financial systems.

### 9.4. Scope Conditions and Generalisation

The focus on the JSE-Eskom panel is a deliberate identification strategy. Eskom load-shedding stages are documented, exogenous, and time-stamped, with no plausible reverse causality from JSE sector performance to Eskom generation decisions at the daily frequency [37]. This provides unusually clean causal identification of the stress-transmission channel relative to the broader financial contagion literature [1], where stress shocks are typically endogenous to financial system behaviour.

Two requirements must be satisfied for the framework to apply in other markets. The theoretical requirement is a documented exogenous shock series sufficient to define the stressed versus resting distributions. This condition is satisfied in any market with an identifiable external stress event, including sovereign credit events, natural disasters, and major regulatory announcements. The empirical requirement is a panel of N≥30 securities with T≥500 trading days to support KSG estimation in d=6 dimensions with the minimax-optimal $k^{*}\approx T_{0}^{7/9}$ (Theorem 2). This is readily satisfied by most major emerging market exchanges.

Candidate markets for replication include the Nigeria Stock Exchange (NGX), the Nairobi Securities Exchange (NSE), and the Egyptian Exchange (EGX), all of which have experienced documented infrastructure or regulatory stress events. In developed markets, the central bank intervention dates or natural disaster events could serve as exogenous shocks. The deep neural framework deployed here has already been extended to cryptocurrency markets [30] and to JSE-Top40 forecasting [29], confirming that the information-geometric attribution approach generalises across asset classes and market structures. Cross-market comparison would also allow an assessment of whether the energy-to-financials STIF ratio of 4.7 during Stage 6 events is specific to the South African infrastructure context or reflects a more general pattern of utility-sector stress propagation.

### 9.5. Limitations

**Conjecture rather than theorem.** The Entropy–Saliency Equivalence is presented as an empirically verified conjecture because the Fisher-score bridge of Lemma 2 relies on a first-order approximation. The $O({∥\Delta \theta ∥}^{2})$ remainder in Equation (13) is bounded but not eliminated. Elevating the result to a theorem would require either a stronger regularity condition on the PMNet architecture or a non-asymptotic bound on the remainder that is uniform over the JSE panel. Both are directions for future theoretical work. In this respect, the present paper occupies the same position as early transfer entropy results before the consistency proof of Kozachenko and Leonenko [26]: empirically well-supported with a clear theoretical pathway to a complete proof.

**Exponential family assumption.** The Gaussian exponential family assumed in the empirical implementation may be misspecified for the 16 high-kurtosis securities identified in Section 8.9. The three-part empirical justification of Section 7.2—residual kurtosis reduction (0.83 versus raw 6.83), Gaussian AIC weight (0.73 mean across 71/87 securities), and a positive Fisher eigenvalue (${\hat{\lambda }}_{\min}=0.14$) establishes the assumption is well-supported for the majority of the panel. The Student-*t* robustness analysis (${\hat{\rho }}_{S}=0.941$ energy, 0.917 materials) confirms that this misspecification does not materially affect attribution rankings, but researchers working with panels that have heavier residual tails should consider the Student-*t* exponential family or the non-parametric KL estimator of Wang et al. [47] as alternatives. The distinction from prior work is important: whereas Cont [2] documented heavy tails as a stylised fact of financial returns, the present paper shows that the entropic loss function of the GWS-STNet effectively removes the heavy-tailed component from the PMNet residuals for 71/87 JSE securities, making the Gaussian assumption empirically defensible in this specific architectural context.

**Transfer entropy stationarity.** The TE estimates are computed once on the training baseline and held fixed. The rolling-window analysis confirms stability for 2019–2026, but the COVID-19 exception (${\hat{\rho }}_{S}=0.76$) is a reminder that extraordinary structural breaks can alter the Granger causal network of financial sectors. This limitation is shared by all TE-based financial network studies, including Dimpfl and Peter [19] and Sandoval [20], neither of which addresses the stationarity of their TE estimates over multi-year evaluation windows. The paper quantifies this limitation explicitly through the rolling-window Spearman analysis, which is a methodological advance over simply assuming stationarity.

**Single market validation.** All empirical results are from the JSE panel. While the theoretical framework is market-agnostic and the scope conditions of Section 9.4 are broadly satisfied by other emerging markets, formal out-of-market validation remains a priority for future work. Kwon and Yang [18] demonstrated cross-market TE analysis across Asian stock indices; a similar cross-market design applied to the STIF framework would allow direct comparison of infrastructure stress transmission patterns across emerging economies.

**Computational cost and research demands.** The full N×N KSG transfer entropy matrix requires 14.2 h on 8 CPU cores. While the sparse approximation (top-20 pairs per sector, 1.8 h, Spearman fidelity ${\hat{\rho }}_{S}=0.976$) makes the framework deployable within overnight batch processing capacity, it is not yet suitable for real-time intraday deployment. More broadly, the full implementation of this framework from panel construction through KSG estimation, PMNet training, Jacobian backpropagation, STIF computation, and bootstrap calibration requires a dedicated researcher with concurrent expertise in non-parametric statistics, deep learning, information geometry, and financial econometrics. This is an acknowledged practical limitation: the framework is currently a research instrument rather than a turnkey risk management tool. Translating it into production-grade software accessible to risk officers without specialist training is a non-trivial engineering task and an explicit priority for future work. GPU-accelerated approximate nearest-neighbour methods [28] and sparse TE estimation guided by economic sector linkages [48] are promising directions for reducing the computational burden to near-real-time, which would be required for the framework to meet the intraday monitoring requirements envisaged by the FSRA [3] and the IMF’s guidance on AI in finance [45].

## 10. Conclusions

This paper established the information-theoretic foundation for the Metabolic Saliency attribution metric through four contributions, each grounded in the information geometry of the Power Mapping Network output distribution and validated on a 623-day JSE hold-out panel spanning January 2024 to April 2026.

**The Entropy–Saliency Equivalence** (Conjecture 1) established and empirically verified that normalised Metabolic Saliency $S_{\mathrm{ms}}\left(i,t\right)/C_{\mathrm{TE}}^{\left(i\right)}$ is an asymptotically unbiased estimator of the local KL divergence $\Delta_{t}^{\left(i\right)}=\mathrm{KL}\left(q_{t}^{\left(i\right)}∥q_{0}^{\left(i\right)}\right)$, with bias decaying at $O(T_{0}^{-1})$ under Gaussian regularity conditions. The derivation uses the Fisher score representation of the PMNet Jacobian (Lemma 2), the KL-Fisher approximation (Lemma 1), and the Cramér–Rao lower bound (Theorem 1). The result distinguishes this paper from post hoc attribution methods [5,6,9] by establishing convergence to a quantity with a direct statistical-mechanical interpretation—the information cost of the current market state relative to the homeostatic resting baseline—which is consistent with the financial metabolomics paradigm of the series.

**The KSG bias–variance decomposition** (Theorem 2) characterised the finite-sample properties of the transfer entropy estimator used to construct the saliency weights, establishing a minimax-optimal MSE rate of $O(T_{0}^{-4/9})$ for the JSE panel parameter values ($l^{*}=3$, d=6, s=2) and confirming that k=5 nearest neighbours is the MSE-optimal choice ($k^{*}\approx 5.2$), with STIF rankings robust across k∈{3,5,7} (${\hat{\rho }}_{S}>0.979$). This advance over existing TE network studies [19,20], which applies the KSG estimator without characterising its finite-sample uncertainty, ensures that every STIF value is accompanied by a statistically grounded confidence interval.

**The STIF metric** (Definition 6, Proposition 1) provided a directed, unit-bearing measure of inter-sector stress transmission in bits per trading day, consistent under the equivalence result and directly applicable to regulatory audit. STIF advances beyond the undirected spillover indices of Diebold and Yilmaz [21] and the descriptive TE networks of Kwon and Yang [18] by embedding transfer entropy within a convergent neural attribution framework with formally established uncertainty quantification.

**The three-step regulatory audit protocol** (Section 9.2) operationalised the equivalence result into a procedure aligned with the FSRA [3], MiFID II [4], and IMF guidance on AI explainability in finance [45]. The protocol is distinguished from existing compliance workflows based on SHAP [5] and LIME [6] by its resistance to adversarial manipulation [7] and its grounding in a formally consistent estimator.

Empirical validation confirmed the equivalence ($R^{2}=0.810$, $\hat{\rho }=0.90$, p<0.001, *F*-test p=0.119) on the 623-day hold-out panel (n=54,201 sector-day observations), with the walk-forward $R^{2}=0.789$ and placebo $R^{2}=0.081$ ([0.032,0.121]), confirming that the result is not an artefact of the estimation design.

Asymptotic normality was confirmed (JB=2.31, p=0.315, variance ratio 1.11). The energy sector was identified as the primary stress transmitter during Eskom Stage 4+ events, with aggregate STIF rising from 0.14 bits/day at rest to 0.43 bits/day during Stage 6 (3.1× increase, directional asymmetry ratio 4.7). Robustness to non-Gaussian securities confirmed stability of the main result ($R^{2}=0.815$, slope 0.981, Student-*t* Spearman ${\hat{\rho }}_{S}=0.941$ energy and 0.917 materials), and rolling-window TE analysis confirmed Granger causal network stability (${\hat{\rho }}_{S}=0.91$ over three-year windows, COVID-19 exception ${\hat{\rho }}_{S}=0.76$).

Together, these results address an interconnected set of challenges. **SDG 7** (Affordable and Clean Energy): the information-theoretic cost of Eskom infrastructure failure is quantified in bits per trading day. **SDG 8** (Decent Work and Economic Growth): a regulatory-grade audit protocol supports stable, transparent financial markets. **SDG 9** (Industry, Innovation and Infrastructure): the paper contributes the first information-geometric attribution framework for infrastructure-coupled financial systems, grounded in the Cramér–Rao efficiency bound [14]. **SDG 17** (Partnerships for the Goals): the open Zenodo pipeline [49] enables replication on other emerging market panels without proprietary data.

### Future Directions

Paper 3 of the Financial Metabolomics Series will establish the **Fractal Conservation Law**—the conjecture that total system power $P_{\mathrm{total}}=P_{\mathrm{predict}}+\dot{S}$ is approximately conserved across resolution scales (one-day, five-day, and twenty-two-day rolling windows) in the GWS-PMNet framework—which will be verified via multi-resolution Hurst exponent analysis and wavelet variance decomposition [50]. If established, the conservation law would provide the scaling complement to the topological stability of Paper 1 [10] and the information-theoretic equivalence of this paper, completing the three-pillar financial metabolomics framework. Cross-market validation of the STIF framework on the Nigeria Stock Exchange, Nairobi Securities Exchange, and Egyptian Exchange and quantitative benchmark comparisons of STIF against SHAP and Integrated Gradients are additional priorities for future work.

## Figures and Tables

**Table 2:** Data diagnostics for the information-theoretic analysis. Cross-sectional mean and interquartile range across 87 JSE securities, training baseline ($T_{0}=992$ days, January 2015 to December 2018). Residual kurtosis: computed after GWS-STNet fitting. KS continuity: *p*-value of the Kolmogorov–Smirnov continuity test [38]. Ljung-Box: lag at which squared-return autocorrelation becomes insignificant at the 5% level [39].

Statistic	Mean	P25	P75	Implication
Residual excess kurtosis	0.83	0.41	1.24	Approximately Gaussian residuals
Gaussian AIC weight [40]	0.73	0.61	0.87	Exponential family assumption valid
KS continuity *p*-value	0.41	0.28	0.57	s≥2 density smoothness
Ljung-Box insignificance lag	11	8	15	Mixing; block b=9 justified
KSG bias (LOO, bits)	0.013	0.007	0.019	Less than 5% of TE estimates
Bootstrap CI width (bits)	0.112	0.071	0.154	Acceptable saliency weight precision
${\hat{\lambda }}_{\min}$ Fisher eigenvalue	0.14	0.07	0.31	Assumption 1(ii) empirically verified

**Table 3:** Panel regression: normalised Metabolic Saliency $S_{\mathrm{ms}}\left(i,t\right)/C_{\mathrm{TE}}^{\left(i\right)}$ on estimated KL divergence ${\hat{\Delta }}_{t}^{\left(i\right)}$. N=87 sectors, $T_{\mathrm{test}}=623$ days, n=54,201 observations. Standard errors (in parentheses) two-way clustered by sector and day [41]. Sector and day fixed effects included. *** *p* < 0.001.

Variable	Coef.	(SE)	*t*-Stat	*p*-Value
${\hat{\Delta }}_{t}^{\left(i\right)}$ (slope)	0.974 ***	(0.0059)	165.2	<0.001
Intercept	0.003	(0.0034)	0.91	0.365
$R^{2}$	0.810			
Adj. $R^{2}$	0.809			
Walk-forward $R^{2}$ (days 993–2234)	0.789			
Placebo $R^{2}$ (mean, 50 permutations)	0.081			
*F*-test (β=1,α=0)	F=2.14		p=0.119	
Sector FE	Yes			
Day FE	Yes			

**Table 4:** Comparative attribution benchmarks on the JSE hold-out panel (N=87, $T_{\mathrm{test}}=623$ days). $\hat{\rho }$: Pearson correlation with ${\hat{\Delta }}_{t}^{\left(i\right)}$ (empirical for Metabolic Saliency; qualitative for benchmarks based on theoretical properties). Asymmetry: directional ratio STIF(energy→fin)/STIF(fin→energy) during Stage ≥4 events. Cost: approximate computation time on the JSE panel.

Method	$\hat{\rho }$ vs. ${\hat{\Delta }}_{t}$	Asymmetry	Directional	Cost (h)
Metabolic Saliency/STIF	0.90	4.7	Yes	5.8
Integrated Gradients [9]	lower ^†^	n/a	No	∼1.2
SHAP (TreeExplainer) [5]	lower ^†^	n/a	No	∼0.8
DY-FEVD [21]	lower ^†^	2.1	Yes	∼0.3
Pearson network	lowest ^†^	n/a	No	∼0.1

^†^ Qualitative: Integrated Gradients uses a fixed zero-input baseline inconsistent with the distributional resting state [9]; SHAP requires feature independence [34], which is violated in the JSE panel; DY-FEVD requires a VAR specification that cannot be tied to a KL divergence benchmark [21].

**Table 5:** KSG estimator diagnostics for five representative sector pairs ($l^{*}=3$, k∈{3,5,7}, $T_{0}=992$, d=6). Bias estimated by leave-one-out cross-validation. Spearman ${\hat{\rho }}_{S}$: rank correlation of STIF rankings versus k=5 reference. All values in bits.

Pair (j→i)	$\hat{\mathrm{TE}}$	Bias (k=3)	Bias (k=5)	Bias (k=7)	95% CI	${\hat{\rho }}_{S}$ vs. k=5
Energy → Financials	0.431	0.021	0.013	0.019	[0.35,0.51]	−
Materials → Industrials	0.387	0.019	0.015	0.017	[0.31,0.46]	0.983
IT → ConsDisc	0.213	0.012	0.009	0.011	[0.17,0.26]	0.991
Utilities → Energy	0.089	0.006	0.004	0.005	[0.06,0.12]	0.987
Financials → Materials	0.174	0.010	0.007	0.009	[0.14,0.21]	0.979

## Data Availability

All derived quantities required to reproduce the results are publicly available on Zenodo (https://doi.org/10.5281/zenodo.20058349, v2.0, accessed on 12 May 2026, N=87 securities, T=2857 trading days, 5 January 2015 to 29 April 2026). Raw JSE security-level prices are proprietary and are not redistributed. Code is also available at https://github.com/ntebo40/IST-04-Financial-Metabolomics, accessed on 12 May 2026.
